# Peppers: A “Hot” Natural Source for Antitumor Compounds

**DOI:** 10.3390/molecules26061521

**Published:** 2021-03-10

**Authors:** Micael Rodrigues Cunha, Maurício Temotheo Tavares, Thais Batista Fernandes, Roberto Parise-Filho

**Affiliations:** 1 Center of Medicinal Chemistry, Dr. André Tosello Avenue, 550, Campinas, SP 13083-886, Brazil; micaelrc@unicamp.br; 2 Laboratory of Design and Synthesis of Bioactive Substances, Department of Pharmacy, University of São Paulo, Prof. Lineu Prestes Avenue 580, Bl.13, Butantã, SP 05508-900, Brazil; mttavares@scripps.edu (M.T.T.); thaisbf@alumni.usp.br (T.B.F.); 3 Department of Molecular Medicine, The Scripps Research Institute, Jupiter, FL 33458, USA

**Keywords:** peppers, *Piper*, *Capsicum*, secondary metabolites, antitumor activity, apoptosis

## Abstract

*Piper*, *Capsicum*, and *Pimenta* are the main genera of peppers consumed worldwide. The traditional use of peppers by either ancient civilizations or modern societies has raised interest in their biological applications, including cytotoxic and antiproliferative effects. Cellular responses upon treatment with isolated pepper-derived compounds involve mechanisms of cell death, especially through proapoptotic stimuli in tumorigenic cells. In this review, we highlight naturally occurring secondary metabolites of peppers with cytotoxic effects on cancer cell lines. Available mechanisms of cell death, as well as the development of analogues, are also discussed.

## 1. Introduction

Antineoplastic chemotherapy remains a challenge nowadays since the current drugs affect both tumorigenic and healthy cells, causing undesirable adverse effects due to low selectivity and high toxicity [1]. Moreover, resistance against anticancer drugs may brutally impair the effectiveness of chemotherapy. These issues illustrate the need for new anticancer therapies and the development of more effective and safer antitumor agents [2].

Natural products play an important role in the discovery of new drugs and in addition, they are an important source of innovative molecular scaffolds for the treatment of various diseases, especially cancer. According to Newman and Cragg (2016) [3], among antitumor drugs approved worldwide between 1940 and 2014, 49% of the new molecular entities were natural products or directly derived compounds. Big pharmaceutical companies have retreated from their natural product-derived drug discovery projects, yet several authors have reported new methods and techniques that enhance exploration of the chemical diversity of natural products (e.g., mass spectrometry, genomics, proteomics, automated extract production, and phenotypic high-throughput screening) [3,4,5,6,7,8]. Of note is that these new techniques have allowed the identification of many active compounds in traditional medicines [9,10,11,12,13,14,15].

Primarily used as spices for foods due to the pungent flavor and aroma, peppers have an important position as excellent producers of secondary metabolites that have a wide range of pharmacological properties. For instance, the *Piper*, *Capsicum*, and *Pimenta* genera have been used by ancient civilizations (e.g., Chinese, Mayan, and Caribbean traditional medicines) in formulations for cancer treatment. However, their value as a natural source for cytotoxic compounds has only gained attention in the last decades [16,17,18,19]. Herein, we summarize the in vitro proapoptotic activity of secondary metabolites of peppers and discuss the current efforts to produce pepper-derived analogues with enhanced cytotoxic activity. We observed that most of the research in this field was done by academic institutions. Although many compounds have a potent proapoptotic profile, high selectivity for cancer cells, and easy synthetic accessibility, none of them have progressed to the clinics so far.

## 2. Pepper Ethnopharmacology

Piperaceae, a promising natural source for new drugs, is a pantropical family of plants comprising approximately 4000 species that contain biologically active natural products, including amides, lignans, neolignans, benzopyrene, pyrones, flavonoids, and terpenoids. These compounds led peppers to be broadly used in folk medicine worldwide, especially in Asia and Latin America [16,20,21]. The Piperaceae family has five genera: *Macropiper*, *Zippelia*, *Peperomia*, *Manekia*, and *Piper*, which is the largest genus of this family (nearly 2000 species) [22]. Many *Piper* species are popularly used for the treatment of several disorders, such as rheumatism [23], cardiac arrhythmias [24], asthma [25], upset stomach [26], and many kinds of infections [21]. Further biological properties have been reported for secondary metabolites of *Piper*, such as antinociceptive [27], anti-inflammatory [28,29], antiplatelet aggregation [30], antioxidant [31], antiophidic [32], anxiolytic/antidepressant [33], antidiabetic [32], hepatoprotective [34], leishmanicidal [35], anti-secretory [36], and cytotoxic effects [37].

The Solanaceae family comprises 98 genera and nearly 2700 species [38]. Interestingly, common dietary ingredients appear in Solanaceae subfamilies, such as tomatoes and potatoes (*Solanum*), bell and chili peppers (*Capsicum*), and tobacco (*Nicotiana*) [39]. The biological aspects of this family are primarily related to their alkaloid content (e.g., tropanes, nicotine, capsaicinoids, and glycoalkaloids) [40,41,42,43,44,45]. Chili peppers that are found in the *Capsicum* genus are believed to have been part of the human diet since immemorial time. It is well established that Central and South American Indians grew these peppers before Christopher Columbus’ arrival [46]. The *Capsicum* genus comprises ~27 species with a large number of varieties [47,48]. Among the related biological activities, chili peppers are believed to act as antioxidants [49,50] and hypoglycemic [51], antimicrobial [12], anti-inflammatory [52], thermoregulatory [53], and antitumor [54] agents. 

According to several authors [55,56], the Myrtaceae family is composed of 5500 species that are clustered into 140 genera that are widely distributed in neotropical forests and savannas. This massive family is widely explored for the production of essential oils and spices (*Myrtus* sp. and *Pimenta* sp.) [57,58], in natura food [59], and wood-derived products (*Eucalyptus* sp.) [60]. The *Pimenta* genus comprises 16 species mainly found in the Caribbean region [55,61,62], and its essential oil and leaf extracts have several biological properties such as cytotoxicity [63], anti-nociceptive and anti-inflammatory [64,65], antioxidant [66,67], insecticidal [68], antimicrobial [69,70], and antifungal [71] effects.

## 3. The Apoptosis Pathways

Apoptosis, a programmed senescence process of cell death, naturally occurs (i) when cells lose their proliferative capacity after a certain number of cell divisions, (ii) in cellular defense events (e.g., immune reactions), and (iii) and after severe cellular damage (e.g., solar radiation) [72,73]. Nevertheless, apoptosis can be avoided due to deregulation of extrinsic and intrinsic key components that trigger its pathway, a very common characteristic in many cancers [74]. Advances in the understanding of these biochemical pathways have created opportunities to modulate defective processes through the proapoptotic activity induced by natural and synthetic compounds [75,76].

Most known proapoptotic effects act as upregulation of death receptors, leading to activation of caspases and cell death (via extrinsic pathway) [77,78]. On the other hand, the intrinsic pathway can be triggered by compounds that generally produce high levels of damaged DNA [79]. These compounds, natural or synthetic, can also stimulate proapoptotic regulators of the B-cell lymphoma 2 (BCL-2) family [80], promoting the collapse of internal mitochondrial membrane potential (Δψ) followed by an overflow of the mitochondrial content, such as cytochrome *c* (Cyt *c*), direct IAP binding protein with low pI), and HtrA2 (High temperature requirement protein A2 (DIABLO) [81,82]. In the cytosol, Cyt *c* forms the apoptosome, which promotes the activation of caspases, resulting in apoptosis [83,84].

Among the reviewed compounds, the secondary metabolites of peppers, some analogues, and their potency over cancer cell lines are described in Table 1 and Table S1. Moreover, as can be seen in the next items of this review, chemical constituents are described in detail and cell death mechanisms, when available, are also presented.

## 4. Literature-Related Cytotoxic Compounds

### 4.1. Piper *sp.*

**Figure 1:**
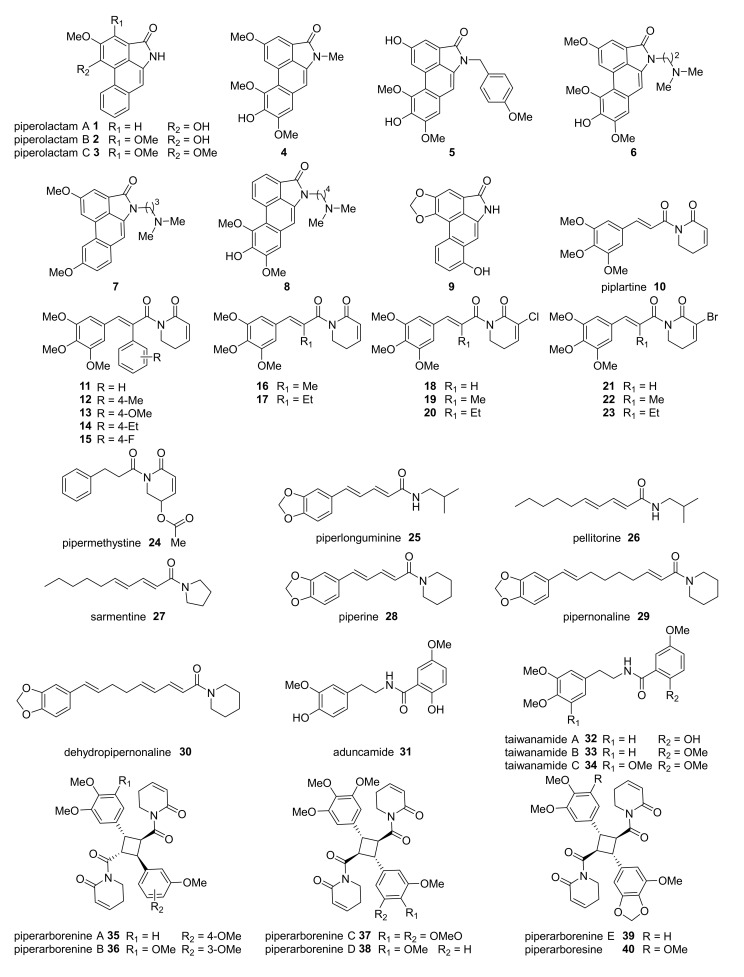

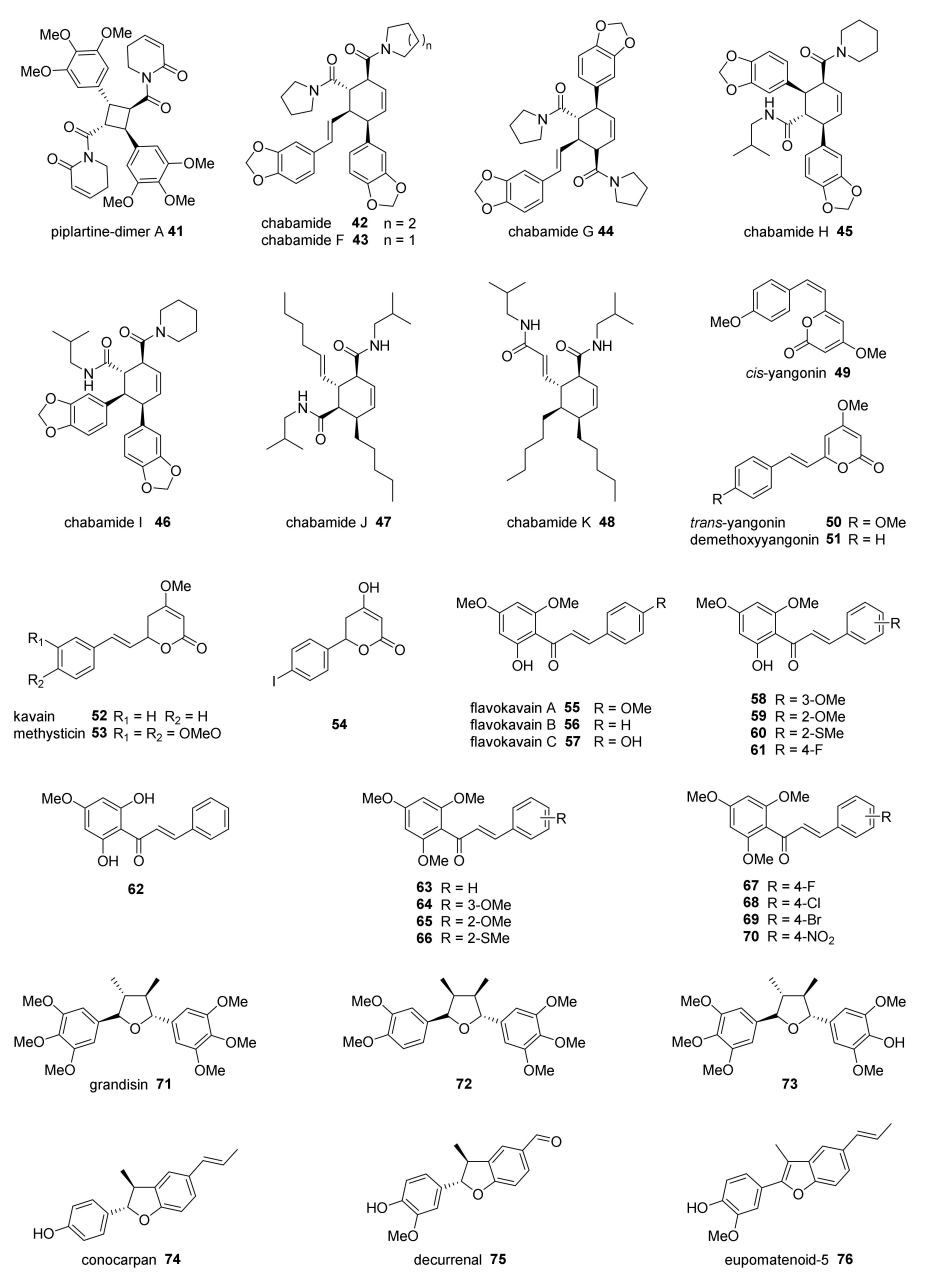
Chemical structures of the reported *Piper* sp. cytotoxic compounds and analogues.

Piperolactams **1–3** (Figure 1) are present in several species of *Piper*, such as *P. caninum*, *P. marginatum*, and *P. kadsura* [98,138,139]. This class of compounds is metabolized in vitro and in vivo to a reactive cyclic *N*-acylnitrenium ion that forms DNA adducts with purine bases, leading to cancer cell death; however, genotoxic and carcinogenic effects in non-tumorigenic cells were observed, as well as shrimp and mice toxicity [140,141,142]. Compounds **1–2** demonstrated moderate (IC_50_ ~10.0 µM) cytotoxicity against A549 lung and SK-MEL-2 skin cancer cells [139,141], whereas **3** was weakly active against P-388 lymphoma and HT-29 adenocarcinoma cells [85]. Many analogues of **1–3**, based on different substitutions at the aristolactam and aporphine moieties, were also achieved. In 2002, Couture et al. (2002) observed that changes in the hydroxyl and methoxyl substituents conferred potent compounds against L1210 leukemia cells in the low µM range (**4–8**, Table 1) [87]. Hedge and coworkers (2010) evaluated the activity of semi-synthetic aristolactams against CDK2, a kinase protein involved in cell cycle regulation. The most potent analogue found (**9**) displayed strong CDK2 inhibition (IC_50_ = 35 nM) and cytotoxicity against MCF-7 breast cancer cells (IC_50_ = 2.0 μM) [89].

Piplartine or piperlongumine **10** is the major bioactive alkaloid extracted from the dried fruits of the *Piper* genus [143,144], of which the species *P. longum* L., *P. tuberculatum*, and *P. chaba* are the most prominent [145]. The literature correlates the observed cytotoxicity of **10** against tumorigenic and normal cell lines (Table 1) to an accumulation of Reactive Oxygen Species (ROS) due to the interaction with antioxidant proteins, activation of p38, and c-Jun *N*-terminal kinases (JNKs), thus leading to cell damage and apoptosis [146,147]. Many compounds derived from **10** were synthesized and evaluated against cancer cell lines. Curiously, the insertion of aryl and alkyl groups to the cinnamyl moiety (**11–23**) afforded potent compounds against A549 lung and MCF-7 and MDA-MB-231 breast cancer cells. Replacement of the acidic proton from the di-hydropyridinone moiety by halogens (**18–23**) also generated cytotoxic compounds [88,93]. An interesting review regarding analogues of **10**, as well as their anticancer properties and molecular bases for their activity, was written by Piska and coworkers [148].

Pipermethystine **24** is another important alkaloid with antitumor activity, which was isolated from leaves of *P. methysticum* [149] and, subsequently, Nerurkarand et al. (2004) observed that **24** inhibited 90% of cellular viability in HepG2 liver carcinoma cells at 100 μM. It is interesting to note that the inhibitory effect of **24** caused a mitochondrial disruption, reduction of adenosine triphosphate (ATP) concentrations, and activation of caspase-3, leading to apoptosis [73,78,95].

Piperlonguminine **25**, found in *P. divaricatum*, *P. longum*, *P. ovatum*, and also in other *Piper* species, was recently patented due to its cytotoxic properties against cancer cells [150,151]. Compound **25** demonstrated potent proapoptotic activity against breast cancer cells by activation of caspases-3, -7, -8, the BAX protein, and the induction of cell cycle arrest at the G_2_/M phase with a reduction in topoisomerase II expression, leading to DNA damage [96,152].

Pellitorine **26** and sarmentine **27** are found in several *Piper* species, such as *P. tuberculatum*, *P. nigrum*, *P. sintenense*, *P. sarmentosum*, *P. nigrum*, and *P. lolot* [21,99,153]. Compound **26** was found to be cytotoxic towards MCF-7 breast cancer cells (IC_50_ = 8.0 µM) and HL60 human leukemia (IC_50_ = 58.0 µM), whereas **27** was only found to be active against P-388 leukemia cells (ED_50_ = 13 µM) [98].

Piperine **28** is the major alkaloid found in *P. nigrum*, the most common pepper species used as a spice in almost every culture worldwide [154]. The cytotoxic activity of **28** was evaluated against several cancer cells and caused the induction of cell cycle arrest at the G_2_/M phase, the activation of caspase-3 and -9, an increase in BAX, and a concomitant reduction in BCL-2 (mediated by p53). Additionally, **28** caused upregulation on the expression of TRPV1 receptors, MMP-2, and MMP-13 [102]. An interesting review about the structure–activity relationship regarding analogues of **28** was reported by Qu et al. [155].

Pipernonaline **29** and dehydropipernonaline **30** were isolated from fruit extracts of *P. retrofractum* [103,156] and *P. longum* L. [157,158]. Both **29** and **30** revealed promising cytotoxic activity against L5178Y mouse lymphoma and PC-3 human prostate cancer cells by inducing cell cycle arrest at the G_0_/G_1_ phase, caspase-3 activation, ROS production, and mitochondrial membrane disruption [103,159].

Aduncamide **31** was first isolated from the leaves of *P. aduncum* as part of a Swiss research program interested in the isolation of biologically active metabolites found in the traditional medicine of Papua New Guinea [104,105]. Even though **31** presented cytotoxicity against KB cells (HeLa-derived tumorigenic cells, ED_50_ = 18.0 µM), no further research was conducted with this compound. Although three natural analogues of **31** were found in *P. taiwanense* (**32–34**), no cytotoxicity has been observed for this set of compounds so far [106].

Piperarborenines **35–41** were isolated from *P. arborescens* [160] and demonstrated potent cytotoxic activity against human cancer cells, reaching submicromolar activity [85]. Notably, a remarkable potent activity was found for **39** against P-388 leukemia (IC_50_ = 0.02 µM), HT-29 colon, and A549 lung cells (IC_50_ = 0.20 µM for both cell lines). The chemical complexity of this class of compounds and its promising anticancer activity is highlighted by the number of publications focusing on the synthesis of **39–40** and related analogues [94,161,162,163,164].

Chabamides **42–48** have been isolated from *P. chaba* [165] and are naturally produced by the condensation of **28** with further secondary metabolites [166] via the Diels–Alder reaction [107]. Compound **42** presented proapoptotic effects in cancer cell lines, inducing cell cycle arrest at the G_0_/G_1_ phase, increased p21 and BAX, and decreased BCL-2 antiapoptotic proteins [108,167]. Compounds **43–48** were found to be less active than **42**, in which remarkable proapoptotic activity was found towards COLO-205 colon cancer cells (IC_50_ = 36.9 nM) [107].

The cytotoxic compounds **49–53** were discovered on *P. methysticum*, a largely consumed spice in Pacific cultures [168,169]. Curiously, the cis-pyranone **49** was threefold more cytotoxic towards K652 leukemia cells than the trans isomer **50** (IC_50_ = 1.6 and 5.5 µM, respectively) [109]. The mechanism of apoptosis was studied in HepG2 liver cancer cells in which chromatin condensation and nuclear fragmentation were observed [170]. Further derivatives of **52** have been evaluated against tumorigenic cells. The most active compound of the series (**54**), however, presented twofold higher cytotoxicity for human normal keratinocytes than for melanoma cells, impairing further studies in vivo [110]. Moreover, compounds **49–53** were also reported to be potent cytochrome P450 inhibitors and hepatotoxic [171].

Chalcones **55–56** were found in *P. methysticum*, *P. dilatatum*, and *P. rusbyi* [109,172,173]. Even though these compounds were strongly associated with death receptor upregulation [115,116], further studies suggested that along with **57**, they might modulate the BLC-2 family, inducing mitochondrial disruption and downregulation of X-linked inhibitor of apoptosis protein (XIAP) [119,174,175]. Western blot analysis also indicated the cleavage of Poly (ADP-ribose) polymerase (PARP) mediated by JNK [117], Akt/MAP-kinase inactivation, and a reduction in the levels of cyclin A and B1, Cdc2, and Cdc25C [176,177]. Curiously, **56** was highly cytotoxic against HCT116 colon carcinoma and PC-3 prostate cancer cells (IC_50_ = 7.5 and 6.2 µM, respectively), whereas **55** remained inactive [112,113,119,120,178]. Moreover, **56** presented in vivo antitumor activity against DU-145 human prostate cancer and KB cancer cells in tumor xenograft models [113,176]. Analogues **58–70** were evaluated against the liver, colon, breast, prostate, lung, and lymphoma cancer cell lines [112]. Interestingly, the most active compounds were found to be para-substituted by halogens (**67–69**) and nitro (**70**). This set of compounds induced cell cycle arrest at the G_1_/S and M phases, and apoptosis via the PI3K/AKT/mTOR pathway [119,178].

Tetrahydrofuran neolignans such as **71–73** have been isolated from *P. solmsianum*, but they also can be found in species of the Lauraceae, Myristicaceae, and Schisandraceae families [179,180]. Studies have demonstrated that compound **71** has cytotoxic and antitumor activities, suggesting its potential to be used as an anticancer agent [181,182]. Upon treatment with **71**, cancer cells underwent cell cycle arrest at the G_1_ phase, chromatin condensation, phosphatidylserine externalization, DNA fragmentation, upregulation on caspase activity, and apoptosis [122,183]. The poor aqueous solubility of **71** was ameliorated through nanoencapsulation, which presented almost 16-fold higher cytotoxicity against Balb/c 3T3-A31 fibroblasts (IC_50_ = 5.0 nM) [184]. The natural analogue **72** and the demethylated metabolite **73** were also found to be cytotoxic against several cancer cell types [124].

Compounds **74–76** can be found in several species of *Piper*, such as *P. regnellii*, *P. solmsianum*, *P. decurrens*, *P. abutiloides*, *P. kadsurai*, and *P. rivinoides* [185,186,187,188,189,190]. Although **74** was a potent cytotoxic compound over a panel of cancer cells, **75** was slightly active only in MCF-7 breast cancer cells (IC_50_ = 169.1 µM) [125,191]. Moreover, cancer cells treated with neolignan **76** displayed a high apoptosis rate through phosphatidylserine externalization, caspase activation, a loss of cell membrane integrity, and an increase in ROS. Upon treatment with **76**, MCF-7 revealed apoptosis-like alterations such as pyknosis, blebbing, and evagination of plasma membrane; on the other hand, 786-0 cells displayed cytoplasmic content release associated with the necrotic process [192]. Remarkably, in vivo experiments using an Ehrlich solid tumor mice model demonstrated that treatment with **76** reduced the tumor volume by 30% with no observation of adverse effects in mice [127].

### 4.2. Capsicum *sp.*

**Figure 2:**
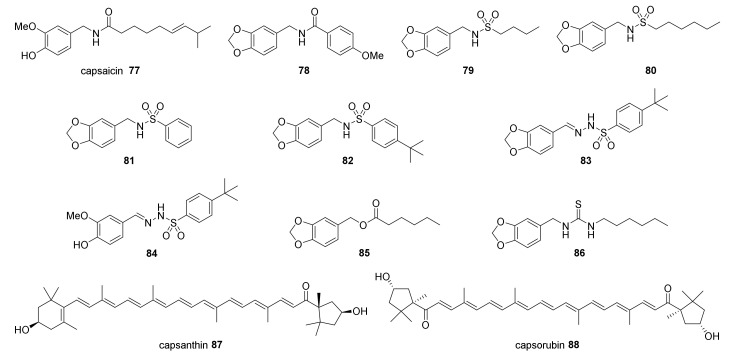
Chemical structures of the reported *Capsicum* sp. cytotoxic compounds and some analogues.

Capsaicinoids are the most studied compounds related to red peppers of the *Capsicum* genus. Jalapeño pepper *(C. annuum*), habanero (*C. chinense*), and tabasco (*C. frutescens*) have a high capsaicinoid concentration, ranging from 0.2% to 4.2% [193,194,195,196], depending on environmental conditions and quantification methods [47]. Capsaicin **77** (Figure 2), is the main capsaicinoid metabolite found in red peppers and can be isolated mainly from fruits of the *Capsicum* species [197]. The analgesic, pungent, and pro-apoptotic effects of **77** are related to their interaction with Transient Receptor Potential Vanilloid (TRPV) receptors at the sensory neurons [198]. This family of transmembrane receptors (TRPV1 to TRPV6) is found in several tissues and mediates the influx of Ca^2+^ into the cytosol [199]. The TRPV receptors can be activated by many stimuli such as proton (H^+^), heat, and natural substances such as **28**, **77,** and resiferatoxin [200,201,202]. In sensory neuronal fibers, the activation of TRPV1 by **77** triggers a rapid increase in Ca^2+^ flux, causing neuronal depolarization and the characteristic burning sensation [203,204,205,206]. Compound **77** is also supposed to interact with other TRP receptors involved in cancer progression, such as TRPV6 [207] and TRPM8 [208]. Chow et al. (2007) [209] suggested that **77** induces apoptosis preferentially via TRPV6, with selectivity for tumor cells. Recently, however, the activity of **77** against TRPV6 was evaluated in a Ca^2+^ flux assay [134,210]. The authors observed that in this assay, the compound was not able to change the channel transport. Despite the mode of action of **77** still being inconclusive, further studies indicated that the modulation of TRP channels and enhancement on Ca^2+^ influx may trigger apoptosis by calpain activation and effector caspases as well [211]. This compound has been investigated against more than 40 types of tumors, attracting the attention of many researchers as a promising drug candidate for cancer treatment [128]. Upon treatment with **77**, tumor cells undergo disruption of the mitochondrial membrane, increasing ROS generation and caspase-3 and -9 activity [212]. In vivo mice models revealed that administration of **77** significantly reduced tumor growth (>50%) in breast and leukemia cancers [76,213]. As the inherent pungency of **77** greatly limits its application in therapeutics, it has led several research groups to design analogues lacking pungency of **77** [129,131,132,133,210,214,215]. 

Compound **78** inhibited MCF-7 breast cancer cells at 32.0 µM, showing a better effect when compared to **77** (53.0 µM). Additionally, common changes typically associated with apoptosis were observed, such as cell shrinkage, pyknosis, mitochondrial depolarization, the formation of apoptotic vesicles, and DNA fragmentation [129]. Furthermore, it was observed that cells treated with **78** exhibited a reduced number of mitoses, disruption of mitotic spindles, and cell cycle arrest at the G_2_/M phase [129]. Compounds **79–82** presented proapoptotic activity against B16F10 murine melanoma and MDA-MB-231 and MCF-7 human breast cancer cells with no pungency in vivo. Moreover, these compounds induced cell cycle arrest and downregulation of BCL-2 expression [129,131]. Noteworthy, **79** significantly reduced tumor volume in a breast tumor model in vivo [129,131]. Further bioisosteric analogues **83–86** exhibited weaker activity over breast cancer cells [130,133,134].

Carotenoids such as **87** and **88** are abundant in red peppers such as *C. annuum*, *C. baccatum*, *C. chinense*, and *C. pubescens* [216]. Compound **87**, in a concentration-independent way, partially reduced prostate cancer cell proliferation, inducing cell cycle arrest and apoptosis, but the effect was less pronounced in vivo using F344 rats [135,217]. On the other hand, compound **88** presented potent cytotoxicity against A549 lung cancer cells, with an IC_50_ < 20.0 µM [136].

### 4.3. Pimenta *sp.*

**Figure 3:**
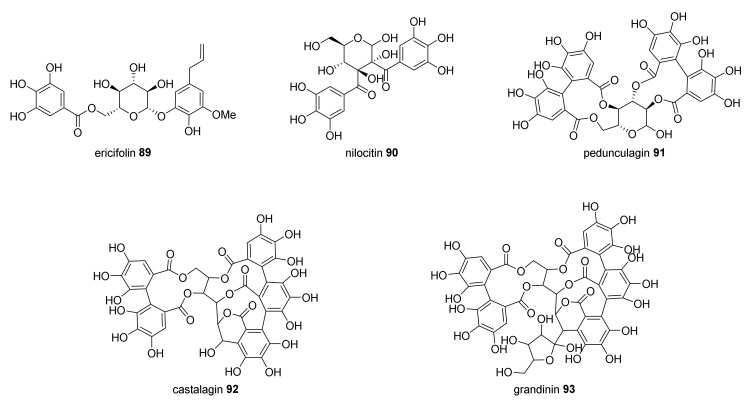
Chemical structures of the reported *Pimenta dioica* cytotoxic compounds.

Amongst the other reviewed genus, *Pimenta* sp. is less explored and possesses fewer representatives (16 species). The cytotoxic compounds related to *Pimenta* sp. reported in the literature came from treatments with extracts of *Pimenta dioica* berries and leaves [19]. Curiously, breast cancer cells underwent autophagy, whereas prostate cancer cells underwent cycle arrest at the G_1_/S phase and also apoptosis. The proapoptotic activity of the extract was linked to the presence of glycopyranoside **89** (Figure 3), which induced apoptosis in LNCaP human prostate adenocarcinoma cells (IC_50_ < 5.0 µM) by reducing cyclin-D1, CDK4, and androgen receptor transcription [15,137]. However, the purified **89** has no activity against MCF-7 and MDA-MB-231 breast cancer cells [137]. Several cytotoxic polyphenols (**90–93**) isolated from *P. dioica* leaves were evaluated in further studies. These compounds were tested against MCF-7 breast, HepG2 liver, and HCT116 colon cancer cells (Table 1) [63]. Compound **91** was the most cytotoxic (IC_50_ = 18.4, 6.4, and 4.4 µM, respectively), presenting the most protective activity against ROS and nitric oxide (NO) release.

## 5. Conclusions

Peppers produced by the *Piper*, *Capsicum*, and *Pimenta* genera are consumed worldwide and represent a significant natural source of secondary metabolites with high chemical diversity. In the last two decades, natural pepper compounds have been inspiring academic and industry researchers due to their cytotoxic effects on many tumorigenic cell lines. This fact highlights the potential of peppers to be used as a natural source of new molecular entities with anticancer activity. However, despite all efforts, antitumor therapy still does not have pepper-derived representatives. We can observe from the literature that compounds such as piperolactams (**1–3**), grandisin (**71**), and capsaicin (**77**) present physical–chemical properties, PK-PD profiles, and/or adverse effects that may impair clinical trials to treat malignancies. Nevertheless, this review has shown several derivatives and analogues with enhanced biological data, with some of them still undergoing preclinical trials and translational research. Of note is that some pepper-derived compounds, for instance, piperarborenines (**35–40**), methysticin (**53**), conocarpan (**74**), and ericifolin (**89**), have an intriguing proapoptotic mechanism but there is still a lack of information on their detailed mechanisms of cell death. This fact shows a promising area of research in *Piper*, *Capsicum*, and *Pimenta* metabolites that can contribute to the design of new chemical entities based on natural scaffolds.

## Figures and Tables

**Table 1:** Potency (IC_50_; µM) of pepper-derived compounds against several cancer cell lines ^1^.

Compound	Cell Line and IC_50_ (µM)	References
Piperolactam A (**1**)	A549 (10.1); HCT15 (27.8); SK-MEL-2 (18.3); SK-OV-3 (18.3)	[85,86]
Piperolactam B (**2**)	A549 (21.7); HCT15 (21.3); SK-MEL-2 (11.6); SK-OV-3 (14.4); P-388 (46.1)	[85,86]
Piperolactam C (**3**)	A549 (>162.0); P-388 (78.0); HT-29 (69.0)	[85]
**4**	L1210 (1.6)	[87,88]
**5**	L1210 (2.6)	[87,88]
**6**	L1210 (2.3)	[87,88]
**7**	L1210 (1.6)	[87,88]
**8**	L1210 (1.8)	[87,88]
**9**	MCF-7 (2.0)	[89]
Piplartine or Piperlongumine (**10**)	518A2 (2.6); A2780 (0.5); A549 (1.9); CEM (4.4); GBM10 (3.8); HCT116 (6.0); HCT8 (2.2); HL60 (5.3); HT1080 (3.4); HT-29 (1.4); JURKAT (5.3); K-562 (5.7); KB (5.6); MCF-7 (5.0); MOLT-4 (1.7); MRC-5 (35.0); SF188 (3.9); SKBR3 (4.0); T98G (4.9); WI38 (26.8); ZR-75-30 (5.9)	[88,90,91,92,93,94]
**11**	A549 (4.1); MCF-7 (4.2)	[88]
**12**	A549 (4.7); MCF-7 (4.9)	[88]
**13**	A549 (1.8); MCF-7 (1.6)	[88]
**14**	A549 (2.0); MCF-7 (1.8)	[88]
**15**	A549 (3.8); MCF-7 (5.0)	[88]
**16**	A549 (24.0); MDA-MB-231 (11.7)	[93]
**17**	A549 (18.0); MDA-MB-231 (23.7)	[93]
**18**	A549 (19.8); MDA-MB-231 (6.7)	[93]
**19**	A549 (3.9); MDA-MB-231 (6.1)	[93]
**20**	A549 (4.1); MDA-MB-231 (7.3)	[93]
**21**	A549 (4.8); MDA-MB-231 (2.7)	[93]
**22**	A549 (2.7); MDA-MB-231 (2.5)	[93]
**23**	A549 (2.2); MDA-MB-231 (2.1)	[93]
Pipermethystine **24**	HepG2 (not reported)	[95]
Piperlonguminine **25**	MCF-7 (6.0); MCF-12A (50.8); MDA-MB-231 (261.7); MDA-MB-468 (8.0); SW-620 (16.9)	[96]
Pellitorine **26**	HL60 (58.0); MCF-7 (8.0)	[97,98]
Sarmetine **27**	P-388 (ED_50_ = 13.0)	[99]
Piperine **28**	A549 (427.5); COLO-205 (46.0); HeLa (95.0); Hep-G2 (70.0); IMR-32 (89.0); MCF-7 (99.0)	[100,101,102]
Piperninaline **29**	L5178Y (17.0)	[103]
Dehydropiperninaline **30**	L5178Y (8.9)	[103]
Aduncamide **31**	KB (ED_50_ = 18.0)	[104,105]
**32**	Not active	[106]
**33**	Not active	[106]
**34**	Not active	[106]
Piperarborenine A **35**	A549 (4.23); HT-29 (6.21); P-388 (0.21)	[85]
Piperarborenine B **36**	A549 (1.39); HT-29 (2.41); P-388 (0.13)	[85]
Piperarborenine C **37**	A549 (0.23); HT-29 (0.26); P-388 (0.18)	[85]
Piperarborenine D **38**	A549 (0.28); HT-29 (0.35); P-388 (0.20)	[85]
Piperarborenine E **39**	A549 (0.19); HT-29 (0.22); P-388 (0.02)	[85]
Piperarboresine **40**	A549 (5.01); HT-29 (5.69); P-388 (4.87)	[85]
Piplartine-dimer A **41**	P-388 (8.48)	[85]
Chabamide **42**	A549 (67.3); CNE (67.0); COLO-205 (5.4); DU-145 (16.0); HeLa (24.0; 189.8); HepG2 (60.8); K-562 (10.8); MCF-7 (39.1); SGC-7901 (12.0)	[107,108]
Chabamide F **43**	COLO-205 (181.7); HeLa (119.4); HepG2 (44.6); HT-29 (259.7); MCF-7 (49.9)	[107]
Chabamide G **44**	COLO-205 (0.0369); HeLa (85.3); HepG2 (108.0); MCF-7 (51.4)	[107]
Chabamide H **45**	COLO-205 (69.5); HepG2 (253.5); MCF-7 (319.4)	[107]
Chabamide I **46**	COLO-205 (80.5); HeLa (263.4)	[107]
Chabamide J **47**	HT-29 (450.4)	[107]
Chabamide K **48**	COLO-205 (379.4); Hela (191.0); HepG2 (437.2); HT-29 (397.8)	[107]
*cis*-Yangonin **49**	A2780 (2.9); K652 (1.6)	[109]
*trans*-Yangonin **50**	A2780 (9.3); K652 (5.5)	[109]
Demethoxyyangonin **51**	A2780 (16.6); K652 (12.6)	[109]
Kavain **52**	A2780 (11.0); K652 (23.2)	[109]
Methysticin **53**	A375 (65.0); HaCaT (29.0)	[110]
**54**	A375 (65.0); HaCaT (29.0)	[110]
Flavokavain A **55**	MCF-7 (25.0); MDA-MB-231 (17.5)	[111,112]
Flavokavain B **56**	A2058 (18.3); ACC-2 (4.7); CaCo-2 (9.9); Cal-27 (26.7); DU-145 (3.9); H460 (18.2); HaCaT (13.6); HCT116 (7.5); HuH7 (15.9); HSC-3 (17.2); LAPC4 (32.0); LNCaP (48.3); MCF-7 (38.4); MCF-7/HER2 (13.6); MDA-MB-231 (12.3/45.0); NCI-H727 (11.3); PC-3 (6.2); RL (8.2); SKBR3/HER2 (10.0); SK-LMS-1 (4.4)	[112,113,114,115,116,117,118]
Flavokavain C **57**	A549 (40.3); CaSKi (39.9); CCD-18Co (160.9); EJ (8.3); HCT116 (12.7); HepG2 (60.0); HT-29 (39.0); L-02 (57.0); MCF-7 (47.6); RT-4 (1.5)	[119,120]
**58**	CaCo-2 (10.0); HaCaT (10.9); HCT116 (9.2); MCF-7 (10.5); NCI-H727 (11.0); PC-3 (9.6); RL (10.1)	[112]
**59**	CaCo-2 (11.2); HaCaT (10.4); HCT116 (7.7); HuH7 (15.0); MCF-7 (10.3); MDA-MB-231 (13.2); NCI-H727 (14.8); PC-3 (7.3); RL (9.0)	[112]
**60**	CaCo-2 (9.6); HaCaT (10.5); HCT116 (10.0); HuH7 (16.6); MCF-7 (15.9); NCI-H727 (9.9); PC-3 (8.7); RL (8.9)	[112]
**61**	CaCo-2 (9.2); HCT116 (12.4); MCF-7 (8.8); PC-3 (13.2); RL (5.4)	[112]
**62**	HCT116 (54.1); MCF-7 (7.3);	[121]
**63**	CaCo-2 (5.8); HaCaT (7.2); HCT116 (6.9); HuH7 (15.5); MCF-7 (9.4); MDA-MB-231 (12.9); NCI-H727 (11.4); PC-3 (5.1); RL (6.9)	[112]
**64**	CaCo-2 (3.9); HaCaT (5.3); HCT116 (4.3); HuH7 (8.9); MCF-7 (9.4); MDA-MB-231 (8.7); NCI-H727 (8.2); PC-3 (3.1); RL (5.9)	[112]
**65**	CaCo-2 (4.5); HaCaT (8.7); HCT116 (4.2); HuH7 (9.8); MCF-7 (8.9); MDA-MB-231 (13.0); NCI-H727 (4.0); PC-3 (8.1); RL (9.0)	[112]
**66**	CaCo-2 (8.8); HaCaT (7.7); HCT116 (6.8); HuH7 (14.1); MCF-7 (9.3); MDA-MB-231 (9.9); NCI-H727 (8.7); PC-3 (7.6); RL (8.3)	[112]
**67**	CaCo-2 (5.5); HaCaT (7.6); HCT116 (6.2); HuH7 (14.6); MCF-7 (7.7); MDA-MB-231 (10.7); NCI-H727 (5.5); PC-3 (5.5); RL (6.4)	[112]
**68**	CaCo-2 (5.7); HaCaT (7.6); HCT116 (5.4); HuH7 (12.7); MCF-7 (7.5); MDA-MB-231 (8.2); NCI-H727 (6.0); PC-3 (5.8); RL (6.5)	[112]
**69**	CaCo-2 (6.8); HaCaT (9.0); HCT116 (6.2); HuH7 (13.9); MCF-7 (9.5); MDA-MB-231 (11.1); NCI-H727 (11.3); PC-3 (7.1); RL (8.3)	[112]
**70**	CaCo-2 (2.6); HaCaT (2.8); HCT116 (2.7); HuH7 (4.9); MCF-7 (5.0); MDA-MB-231 (3.3); NCI-H727 (4.1); PC-3 (2.5); RL (3.4)	[112]
Grandisin **71**	EAT (0.2); HL60 (60.0); U937 (30.0); V79 (174.0)	[122,123]
**72**	A549 (6.90); SK-MEL-2 (4.50); SK-OV-3 (9.40)	[86]
**73**	3T3-A31 (0.043)	[124]
Conocarpan **74**	A549 (11.2); HL60 (5.8); MCF-7 (7.8); SMMC-7721 (8.9); SW-480 (2.1)	[125]
Decurrenal **75**	MCF-7 (169.1)	[126]
Eupomatenoid-5 **76**	786-0 (TGI = 6.6); HT-29 (TGI = 48.5); K-562 (TGI = 338.5); MCF-7 (TGI = 21.2); NCI-H460 (TGI = 34.8); OVCAR-3 (TGI = 18.7); PC-3 (TGI = 21.0); UACC-62 (TGI = 27.9)	[127]
Capsaicin **77**	3T3 (83.0); A375 (6.0); A2058 (200.0); AsPC1 (150.0); B16F10 (117.0); BxPC3 (150.0); HepG2 (50.0); MCF-7 (53.0); MCF-10A H-*ras* (56.0); MDA-MB-231 (21.7); PC-3 (20.0); RT-4 (80.0)	[128,129,130]
**78**	B16F10 (87.0); MCF-7 (32.0)	[128,129,130]
**79**	B16F10 (38.0); MCF-7 (28.0); MDA-MB-231 (87.0)	[131]
**80**	B16F10 (75.0); MDA-MB-231 (109.0)	[132]
**81**	B16F10 (50.0); MCF-7 (32.0); MDA-MB-231 (14.2)	[129]
**82**	B16F10 (120.0); MDA-MB-231 (75.0)	[132]
**83**	MCF-7 (142.4); MDA-MB-231 (104.6)	[133]
**84**	MCF-7 (144.6); MDA-MB-231 (173.2)	[133]
**85**	B16F10 (130.0); SK-MEL-28 (85.0)	[130]
**86**	A2058 (55.2); SK-MEL-25 (67.2); U-87 (86.9)	[134]
Capsanthin **87**	DU-145 (ND); PC-3 (ND)	[135,136]
Capsorubin **88**	A549 (< 20.0)	[135,136]
Ericifolin **89**	LNCaP (< 5.0)	[137]
Nilocitin **90**	HCT116 (19.4); HepG2 (22.8); MCF-7 (40.8)	[63]
Pedunculagin **91**	HCT116 (4.4); HepG2 (6.4); MCF-7 (18.4)	[63]
Castalagin **92**	HCT116 (7.4); HepG2 (9.8); MCF-7 (26.2)	[63]
Grandinin **93**	HCT116 (13.8); HepG2 (18.4); MCF-7 (22.1)	[63]

^1^ IC_50_ = half of maximal inhibitory concentration; ED_50_ = median of effective dose; TGI = total growth inhibition; ND = not determined.

## Data Availability

All data analyzed in this study are included in this article.

## Supplementary Materials

The following are available online: Table S1: Description of cancer cell lines from Table 1.

## References

[1] Pedersen B., Koktved D.P., Nielsen L.L. (2013). Living with Side Effects from Cancer Treatment-A Challenge to Target Information. Scand. J. Caring Sci..

[2] He Q., Shi J. (2014). MSN Anti-Cancer Nanomedicines: Chemotherapy Enhancement, Overcoming of Drug Resistance, and Metastasis Inhibition. Adv. Mater..

[3] Newman D.J., Cragg G.M. (2016). Natural Products as Sources of New Drugs from 1981 to 2014. J. Nat. Prod..

[4] Koehn F.E., Carter G.T. (2005). The Evolving Role of Natural Products in Drug Discovery. Nat. Rev. Drug Discov..

[5] Harvey A.L., Edrada-Ebel R., Quinn R.J. (2015). The Re-Emergence of Natural Products for Drug Discovery in the Genomics Era. Nat. Rev. Drug Discov..

[6] Yao H., Liu J., Xu S., Zhu Z., Xu J. (2017). The Structural Modification of Natural Products for Novel Drug Discovery. Expert Opin. Drug Discov..

[7] Zhang M.M., Qiao Y., Ang E.L., Zhao H. (2017). Using Natural Products for Drug Discovery: The Impact of the Genomics Era. Expert Opin. Drug Discov..

[8] Zhang A., Sun H., Wang X. (2018). Mass Spectrometry-Driven Drug Discovery for Development of Herbal Medicine. Mass Spectrom. Rev..

[9] Chaveerach A., Mokkamul P., Sudmoon R., Tanee T. (2006). Ethnobotany of the Genus *Piper* (Piperaceae) in Thailand. Ethnobot. Res. Appl..

[10] Meghvansi M.K., Siddiqui S., Khan M.H., Gupta V.K., Vairale M.G., Gogoi H.K., Singh L. (2010). Naga Chilli: A Potential Source of Capsaicinoids with Broad-Spectrum Ethnopharmacological Applications. J. Ethnopharmacol..

[11] Khan F.A., Mahmood T., Ali M., Saeed A., Maalik A. (2014). Pharmacological Importance of an Ethnobotanical Plant: *Capsicum Annuum* L.. Nat. Prod. Res..

[12] Cichewicz R.H., Thorpe P.A. (1996). The Antimicrobial Properties of Chile Peppers (Capsicum Species) and Their Uses in Mayan Medicine. J. Ethnopharmacol..

[13] Corson T.W., Crews C.M. (2007). Molecular Understanding and Modern Application of Traditional Medicines: Triumphs and Trials. Cell.

[14] Srinivas C., Sai Pavan Kumar C.N.S., China Raju B., Jayathirtha Rao V., Naidu V.G.M., Ramakrishna S., Diwan P. (2009). V First Stereoselective Total Synthesis and Anticancer Activity of New Amide Alkaloids of Roots of Pepper. Bioorg. Med. Chem. Lett..

[15] Shamaladevi N., Lyn D.A., Shaaban K.A., Zhang L., Villate S., Rohr J., Lokeshwar B.L. (2013). Ericifolin: A Novel Antitumor Compound from Allspice That Silences Androgen Receptor in Prostate Cancer. Carcinogenesis.

[16] Wang Y.-H., Morris-Natschke S.L., Yang J., Niu H.-M., Long C.-L., Lee K.-H. (2014). Anticancer Principles from Medicinal Piper ( Hú Jiāo) Plants. J. Tradit. Complement. Med..

[17] Aggarwal B.B., Ichikawa H., Garodia P., Weerasinghe P., Sethi G., Bhatt I.D., Pandey M.K., Shishodia S., Nair M.G. (2006). From Traditional Ayurvedic Medicine to Modern Medicine: Identification of Therapeutic Targets for Suppression of Inflammation and Cancer. Expert Opin. Ther. Targets.

[18] Caamal-Fuentes E., Torres-Tapia L.W., Simá-Polanco P., Peraza-Sánchez S.R., Moo-Puc R. (2011). Screening of Plants Used in Mayan Traditional Medicine to Treat Cancer-like Symptoms. J. Ethnopharmacol..

[19] Zhang L., Lokeshwar B.L. (2012). Medicinal Properties of the Jamaican Pepper Plant Pimenta Dioica and Allspice. Curr. Drug Targets.

[20] López S.N., Lopes A.A., Batista J.M., Flausino O., Bolzani V.D.S., Kato M.J., Furlan M. (2010). Geranylation of Benzoic Acid Derivatives by Enzymatic Extracts from Piper Crassinervium (Piperaceae). Bioresour. Technol..

[21] Nascimento J.C.d., Paula d.V.F., David J.M., David J.P. (2012). Occurrence, Biological Activities and 13C NMR Data of Amides from Piper (Piperaceae). Química Nova.

[22] Quijano-Abril A., Callejas-Posada R., Miranda-Esquivel D.R. (2006). Areas of Endemism and Distribution Patterns for Neotropical Piper Species (Piperaceae). J. Biogeogr..

[23] Yarnell E. (2017). Herbs for Rheumatoid Arthritis. Altern. Complement. Ther..

[24] Srinivasan K. (2016). Biological Activities of Red Pepper ( Capsicum Annuum ) and Its Pungent Principle Capsaicin: A Review. Crit. Rev. Food Sci. Nutr..

[25] Kim S.-H., Lee Y.-C. (2009). Piperine Inhibits Eosinophil Infiltration and Airway Hyperresponsiveness by Suppressing T Cell Activity and Th2 Cytokine Production in the Ovalbumin-Induced Asthma Model. J. Pharm. Pharmacol..

[26] Mehmood M.H., Gilani A.H. (2010). Pharmacological Basis for the Medicinal Use of Black Pepper and Piperine in Gastrointestinal Disorders. J. Med. Food.

[27] López K.S.E., Marques A.M., Moreira D.D.L., Velozo L.S., Sudo R.T., Zapata-Sudo G., Guimarães E.F., Kaplan M.A.C. (2016). Local Anesthetic Activity from Extracts, Fractions and Pure Compounds from the Roots of Ottonia Anisum Spreng. (Piperaceae). Ann. Braz. Acad. Sci..

[28] Fusco B.M., Giacovazzo M. (1997). Peppers and Pain. The Promise of Capsaicin. Drugs.

[29] Parise-Filho R., Pastrello M., Pereira Camerlingo C.E., Silva G.J., Agostinho L.A., de Souza T., Motter Magri F.M., Ribeiro R.R., Brandt C.A., Polli M.C. (2011). The Anti-Inflammatory Activity of Dillapiole and Some Semisynthetic Analogues. Pharm. Biol..

[30] Park B.S., Son D.J., Park Y.H., Kim T.W., Lee S.E. (2007). Antiplatelet Effects of Acidamides Isolated from the Fruits of Piper Longum L.. Phytomedicine.

[31] Amarowicz R. (2014). Antioxidant Activity of Peppers. Eur. J. Lipid Sci. Technol..

[32] Bezerra D.P., Pessoa C., de Moraes M.O., Saker-Neto N., Silveira E.R., Costa-Lotufo L. (2013). V Overview of the Therapeutic Potential of Piplartine (Piperlongumine). Eur. J. Pharm. Sci..

[33] Cícero Bezerra Felipe F., Trajano Sousa Filho J., de Oliveira Souza L.E., Alexandre Silveira J., Esdras de Andrade Uchoa D., Rocha Silveira E., Deusdênia Loiola Pessoa O., de Barros Viana G.S. (2007). Piplartine, an Amide Alkaloid from Piper Tuberculatum, Presents Anxiolytic and Antidepressant Effects in Mice. Phytomedicine.

[34] Koul I., Kapil A. (1993). Evaluation of the Liver Protective Potential of Piperine, an Active Principle of Black and Long Peppers. Planta Med..

[35] Parise-Filho R., Pasqualoto K.F.M., Magri F.M.M., Ferreira A.K., da Silva B.A.V.G., Damião M.C.F.C.B., Tavares M.T., Azevedo R.A., Auada A.V.V., Polli M.C. (2012). Dillapiole as Antileishmanial Agent: Discovery, Cytotoxic Activity and Preliminary SAR Studies of Dillapiole Analogues. Arch. Pharm. Pharm. Med. Chem..

[36] Pongkorpsakol P., Wongkrasant P., Kumpun S., Chatsudthipong V., Muanprasat C. (2015). Inhibition of Intestinal Chloride Secretion by Piperine as a Cellular Basis for the Anti-Secretory Effect of Black Peppers. Pharmacol. Res..

[37] Ferreira A.K., de-Sá-Júnior P.L., Pasqualoto K.F.M., de Azevedo R.A., Câmara D.A.D., Costa A.S., Figueiredo C.R., Matsuo A.L., Massaoka M.H., Auada A.V.V. (2014). Cytotoxic Effects of Dillapiole on MDA-MB-231 Cells Involve the Induction of Apoptosis through the Mitochondrial Pathway by Inducing an Oxidative Stress While Altering the Cytoskeleton Network. Biochimie.

[38] Olmstead R.G., Bohs L. (2007). A Summary of Molecular Systematic Research in Solanaceae: 1982-2006. Acta Hortic..

[39] Knapp S. (2002). Tobacco to Tomatoes: A Phylogenetic Perspective on Fruit Diversity in the Solanaceae. J. Exp. Bot..

[40] Singh B., Gupta V., Bansal P., Singh R., Kumar D. (2010). Pharmacological Potential of Plant Used as Aphrodisiacs. Int. J. Pharm. Sci. Rev. Res..

[41] Wannang N.N., Anuka J.A., Kwanashie H.O., Gyang S.S., Auta A. (2008). Anti-Seizure Activity of the Aqueous Leaf Extract of Solanum Nigrum Linn (Solanaceae) in Experimental Animals. Afr. Health Sci..

[42] Ndebia E.J., Kamgang R., Nkeh-ChungagAnye B.N. (2007). Analgesic and Anti-Inflammatory Properties of Aqueous Extract from Leaves of Solanum Torvum (Solanaceae). Afr. J. Tradit. Complement. Altern. Med..

[43] Monteiro F.S., Silva A.C.L., Martins I.R.R., Correia A.C.C., Basílio I.J.L.D., Agra M.F., Bhattacharyya J., Silva B.A. (2012). Vasorelaxant Action of the Total Alkaloid Fraction Obtained from Solanum Paludosum Moric. (Solanaceae) Involves NO/CGMP/PKG Pathway and Potassium Channels. J. Ethnopharmacol..

[44] Gandhi G.R., Ignacimuthu S., Paulraj M.G., Sasikumar P. (2011). Antihyperglycemic Activity and Antidiabetic Effect of Methyl Caffeate Isolated from Solanum Torvum Swartz. Fruit in Streptozotocin Induced Diabetic Rats. Eur. J. Pharmacol..

[45] Giorgetti M., Negri G. (2011). Plants from Solanaceae Family with Possible Anxiolytic Effect Reported on 19thcentury’s Brazilian Medical Journal. Braz. J. Pharmacogn..

[46] Govindarajan V.S. (1985). Capsicum-Production, Technology, Chemistry, and Quality Part 1: History, Botany, Cultivation, and Primary Processing. Crit. Rev. Food Sci. Nutr..

[47] Canto-Flick A., Balam-Uc E., Bello-Bello J.J., Lecona-Guzmán C., Solís-Marroquín D., Avilés-Viñas S., Gómez-Uc E., López-Puc G., Santana-Buzzy N., Iglesias-Andreu L.G. (2008). Capsaicinoids Content in Habanero Pepper (Capsicum Chinense Jacq.): Hottest Known Cultivars. HortScience.

[48] Gurnani N., Gupta M., Mehta D., Mehta B.K. (2015). Chemical Composition, Total Phenolic and Flavonoid Contents, and in Vitro Antimicrobial and Antioxidant Activities of Crude Extracts from Red Chilli Seeds (Capsicum Frutescens L.)Gurnani, N.J. Taibah Univ. Sci..

[49] Zhuang Y., Chen L., Sun L., Cao J. (2012). Bioactive Characteristics and Antioxidant Activities of Nine Peppers. J. Funct. Foods.

[50] Oboh G., Puntel R.L., Rocha J.B.T. (2007). Hot Pepper (Capsicum Annuum, Tepin and Capsicum Chinese, Habanero) Prevents Fe2+-Induced Lipid Peroxidation in Brain-in Vitro. Food Chem..

[51] Tundis R., Menichini F., Bonesi M., Conforti F., Statti G., Menichini F., Loizzo M.R. (2013). Antioxidant and Hypoglycaemic Activities and Their Relationship to Phytochemicals in Capsicum Annuum Cultivars during Fruit Development. Lwt Food Sci. Technol..

[52] Zimmer A.R., Leonardi B., Miron D., Schapoval E., Oliveira J.R.D., Gosmann G. (2012). Antioxidant and Anti-Inflammatory Properties of Capsicum Baccatum: From Traditional Use to Scientific Approach. J. Ethnopharmacol..

[53] Govindarajan V.S., Sathyanarayana M.N. (1991). Capsicum—Production, Technology, Chemistry, and Quality. Part v. Impact on Physiology, Pharmacology, Nutrition, and Metabolism; Structure, Pungency, Pain, and Desensitization Sequences. Crit. Rev. Food Sci. Nutr..

[54] De Melo J.G., Santos A.G., De Amorim E.L.C., Nascimento S.C.D., De Albuquerque U.P. (2011). Medicinal Plants Used as Antitumor Agents in Brazil: An Ethnobotanical Approach. Evid. Based Complement. Altern. Med..

[55] Vasconcelos T.N.C., Lucas E.J., Brigido P. (2018). One New Species, Two New Combinations and Taxonomic Notes on the All-Spice Genus Pimenta (Myrtaceae) from Hispaniola. Phytotaxa.

[56] Gomes S.M., Dalla Nora Somavilla N.S., Gomes-Bezerra K.M., de Miranda S.C., De-Carvalhoa P.S., Graciano-Ribeiro D. (2009). Leaf Anatomy of Myrtaceae Species: Contributions to the Taxonomy and Phylogeny. Acta Bot. Bras..

[57] Akin M., Aktumsek A., Nostro A. (2010). Antibacterial Activity and Composition of the Essential Oils of Eucalyptus Camaldulensis Dehn. and Myrtus Communis L. Growing in Northern Cyprus. Afr. J. Biotechnol..

[58] Yokomizo N.K.S., Nakaoka-Sakita M. (2014). Antimicrobial Activity and Essential Oils Yield of Pimenta Pseudocaryophyllus Var. Pseudocaryophyllus (Gomes) Landrum, Myrtaceae. Rev. Bras. Plantas Med..

[59] Weston R.J. (2010). Bioactive Products from Fruit of the Feijoa (Feijoa Sellowiana, Myrtaceae): A Review. Food Chem..

[60] Myburg A.A., Grattapaglia D., Tuskan G.A., Hellsten U., Hayes R.D., Grimwood J., Jenkins J., Lindquist E., Tice H., Bauer D. (2014). The Genome of Eucalyptus Grandis. Nature.

[61] Ramos A., Visozo A., Piloto J., García A., Rodríguez C.A., Rivero R. (2003). Screening of Antimutagenicity via Antioxidant Activity in Cuban Medicinal Plants. J. Ethnopharmacol..

[62] Paula J.A.M., Reis J.B., Ferreira L.H.M., Menezes A.C.S., Paula J.R. (2010). Gênero Pimenta: Aspectos botânicos, composição química e potencial farmacológico. Rev. Bras. Plantas Med..

[63] Marzouk M.S.A., Moharram F.A., Mohamed M.A., Gamal-Eldeen A.M., Aboutabl E.A. (2007). Anticancer and Antioxidant Tannins from Pimenta Dioica Leaves. Z. Nat. C.

[64] Paula J.A.M.D., Silva M.D.R.R., Costa M.P., Diniz D.G.A., Sá F.A.S., Alves S.F., Costa É.A., Lino R.C., Paula J.R. (2012). De Phytochemical Analysis and Antimicrobial, Antinociceptive, and Anti-Inflammatory Activities of Two Chemotypes of Pimenta Pseudocaryophyllus (Myrtaceae). Evid. Based Complement. Altern. Med..

[65] García M.D.,  Fernández M.A., Alvarez A., Saenz M.T. (2004). Antinociceptive and Anti-Inflammatory Effect of the Aqueous Extract from Leaves of Pimenta Racemosa Var. Ozua (Mirtaceae). J. Ethnopharmacol..

[66] Padmakumari K.P., Sasidharan I., Sreekumar M.M. (2011). Composition and Antioxidant Activity of Essential Oil of Pimento (Pimenta Dioica (L) Merr.) from Jamaica. Nat. Prod. Res..

[67] Kikuzaki H., Hara S., Kawai Y., Nakatani N. (1999). Antioxidative Phenylpropanoids from Berries of Pimenta Dioica. Phytochemistry.

[68] Seo S.M., Kim J., Lee S.G., Shin C.H., Shin S.C., Park I.K. (2009). Fumigant Antitermitic Activity of Plant Essential Oils and Components from Ajowan (Trachyspermum Ammi), Allspice (Pimenta Dioica), Caraway (Carům Carvi), Dill (Anethum Graveoiens), Geranium (Pelargonium Graveoiens), and Litsea (Litsea Cubeba) Oils Against. J. Agric. Food Chem..

[69] Enoque M., Lima L., Cordeiro I., Cláudia M., Young M., Sobra M.E.G., Roberto P., Moreno H. (2006). Antimicrobial Activity of the Essential Oil from Two Specimens of Pimenta Pseudocaryophyllus (Gomes) L. R. Landrum (Myrtaceae) Native from São Paulo State–Brazil. Pharmacologyonline.

[70] Saenz M.T., Tornos M.P., Alvarez A., Fernandez M.A., García M.D. (2004). Antibacterial Activity of Essential Oils of Pimenta Racemosa Var. Terebinthina and Pimenta Racemosa Var. Grisea. Fitoterapia.

[71] Zabka M., Pavela R., Slezakova L. (2009). Antifungal Effect of Pimenta Dioica Essential Oil against Dangerous Pathogenic and Toxinogenic Fungi. Ind. Crop. Prod..

[72] Wu C.-C.C., Bratton S.B. (2012). Regulation of the Intrinsic Apoptosis Pathway by Reactive Oxygen Species. Antioxid. Redox Signal..

[73] Ouyang L., Shi Z., Zhao S., Wang F.T., Zhou T.T., Liu B., Bao J.K. (2012). Programmed Cell Death Pathways in Cancer: A Review of Apoptosis, Autophagy and Programmed Necrosis. Cell Prolif..

[74] Hanahan D., Weinberg R.A. (2011). Hallmarks of Cancer: The next Generation. Cell.

[75] Liang X., Xu K., Xu Y., Liu J., Qian X. (2011). B1-Induced Caspase-Independent Apoptosis in MCF-7 Cells Is Mediated by down-Regulation of Bcl-2 via P53 Binding to P2 Promoter TATA Box. Toxicol. Appl. Pharmacol..

[76] Thoennissen N.H., O’Kelly J., Lu D., Iwanski G.B., La D.T., Abbassi S., Leiter A., Karlan B., Mehta R., Koeffler H.P. (2010). Capsaicin Causes Cell-Cycle Arrest and Apoptosis in ER-Positive and -Negative Breast Cancer Cells by Modulating the EGFR/HER-2 Pathway. Oncogene.

[77] Elmore S. (2007). Apoptosis: A Review of Programmed Cell Death. Toxicol. Pathol..

[78] McStay G.P., Green D.R. (2014). Measuring Apoptosis: Caspase Inhibitors and Activity Assays. Cold Spring Harb. Protoc..

[79] Roos W.P., Thomas A.D., Kaina B. (2015). DNA Damage and the Balance between Survival and Death in Cancer Biology. Nat. Rev. Cancer.

[80] Ashkenazi A., Fairbrother W.J., Leverson J.D., Souers A.J. (2017). From Basic Apoptosis Discoveries to Advanced Selective BCL-2 Family Inhibitors. Nat. Rev. Drug Discov..

[81] Luo X., Budihardjo I., Zou H., Slaughter C., Wang X. (1998). Bid, a Bcl2 Interacting Protein, Mediates Cytochrome c Release from Mitochondria in Response to Activation of Cell Surface Death Receptors. Cell.

[82] Vyas S., Zaganjor E., Haigis M.C. (2016). Mitochondria and Cancer. Cell.

[83] Man S.M., Kanneganti T. (2016). Converging Roles of Caspases in Inflammasome Activation, Cell Death and Innate Immunity. Nat. Rev. Immunol..

[84] Lopez J., Tait S.W.G. (2015). Mitochondrial Apoptosis: Killing Cancer Using the Enemy Within. Br. J. Cancer.

[85] Tsai I.-L., Lee F.-P., Wu C.-C., Duh C.-Y., Ishikawa T., Chen J.-J., Chen Y.-C., Seki H., Chen I.-S. (2005). New Cytotoxic Cyclobutanoid Amides, a New Furanoid Lignan and Anti-Platelet Aggregation Constituents from Piper Arborescens. Planta Med..

[86] Kim K.H., Kim H.K., Choi S.U., Moon E., Kim S.Y., Lee K.R. (2011). Bioactive Lignans from the Rhizomes of Acorus Gramineus. J. Nat. Prod..

[87] Couture A., Deniau E., Grandclaudon P., Rybalko-Rosen H., Léonce S., Pfeiffer B., Renard P. (2002). Synthesis and Biological Evaluation of Aristolactams. Bioorg. Med. Chem. Lett..

[88] Punganuru S.R., Madala H.R., Venugopal S.N., Samala R., Mikelis C., Srivenugopal K.S. (2016). Design and Synthesis of a C7-Aryl Piperlongumine Derivative with Potent Antimicrotubule and Mutant P53-Reactivating Properties. Eur. J. Med. Chem..

[89] Hegde V.R., Borges S., Pu H., Patel M., Gullo V.P., Wu B., Kirschmeier P., Williams M.J., Madison V., Fischmann T. (2010). Semi-Synthetic Aristolactams--Inhibitors of CDK2 Enzyme. Bioorg. Med. Chem. Lett..

[90] Chang-Yih D., Yang-Chang W., Shang-Kwei W. (1990). Cytotoxic Pyridone Alkaloids from Piper Aborescens. Phytochemistry.

[91] Bezerra D.P., Pessoa C., Moraes M.O.d., Silveira E.R., Lima M.A.S., Martins Elmiro F.J., Costa-Lotufo L.V. (2005). Antiproliferative Effects of Two Amides, Piperine and Piplartine, from Piper Species. Z. Nat. C.

[92] Bezerra D.P., Militão G.C.G., de Castro F.O., Pessoa C., de Moraes M.O., Silveira E.R., Lima M.A.S., Elmiro F.J.M., Costa-Lotufo L.V. (2007). Piplartine Induces Inhibition of Leukemia Cell Proliferation Triggering Both Apoptosis and Necrosis Pathways. Toxicol. Vitr. Int. J. Publ. Assoc. Bibra.

[93] Wu Y., Min X., Zhuang C., Li J., Yu Z., Dong G., Yao J., Wang S., Liu Y., Wu S. (2014). Design, Synthesis and Biological Activity of Piperlongumine Derivatives as Selective Anticancer Agents. Eur. J. Med. Chem..

[94] Sommerwerk S., Kluge R., Ströhl D., Heller L., Kramell A.E., Ogiolda S., Liebing P., Csuk R. (2016). Synthesis, Characterization and Cytotoxicity of New Piplartine Dimers. Tetrahedron.

[95] Nerurkar P.V., Dragull K., Tang C.-S.S. (2004). In Vitro Toxicity of Kava Alkaloid, Pipermethystine, in HepG2 Cells Compared to Kavalactones. Toxicol. Sci. Off. J. Soc. Toxicol..

[96] Sriwiriyajan S., Sukpondma Y., Srisawat T., Madla S., Graidist P. (2017). (−)-Kusunokinin and Piperloguminine from Piper Nigrum: An Alternative Option to Treat Breast Cancer. Biomed. Pharmacother..

[97] Ee G.C.L., Lim C.M., Rahmani M., Shaari K., Bong C.F.J. (2010). Pellitorine, a Potential Anti-Cancer Lead Compound against HL60 and MCT-7 Cell Lines and Microbial Transformation of Piperine from Piper Nigrum. Molecules.

[98] Gutierrez R.M.P., Gonzalez A.M.N., Hoyo-Vadillo C. (2013). Alkaloids from Piper: A Review of Its Phytochemistry and Pharmacology. Mini Rev. Med. Chem..

[99] Chen J., Duh C., Huang H., Chen I. (2003). Cytotoxic Constituents of Piper Sintenense. Helv. Chim. Acta.

[100] Rao V.R.S., Suresh G., Rao R.R., Babu K.S., Chashoo G., Saxena A.K., Rao J.M., Rama Subba Rao V., Suresh G., Ranga Rao R. (2012). Synthesis of Piperine-Amino Acid Ester Conjugates and Study of Their Cytotoxic Activities against Human Cancer Cell Lines. Med. Chem. Res..

[101] Umadevi P., Deepti K., Venugopal D.V.R. (2013). Synthesis, Anticancer and Antibacterial Activities of Piperine Analogs. Med. Chem. Res..

[102] Lin Y., Xu J., Liao H., Li L., Pan L. (2014). Piperine Induces Apoptosis of Lung Cancer A549 Cells via P53-Dependent Mitochondrial Signaling Pathway. Tumor Biol..

[103] Muharini R., Liu Z., Lin W., Proksch P. (2015). New Amides from the Fruits of Piper Retrofractum. Tetrahedron Lett..

[104] Orjala J., Wright A., Rali T., Sticher O. (1993). Aduncamide, a Cytotoxic and Antibacterial β-Phenylethylamine-Derived Amide from Piper Aduncum. Nat. Prod. Lett..

[105] Rali T., Wossa S.W., Leach D.N., Waterman P.G. (2007). Volatile Chemical Constituents of Piper Aduncum L and Piper Gibbilimbum C. DC (Piperaceae) from Papua New Guinea. Molecules.

[106] Chen I.S., Chen Y.C., Liao C.H. (2007). Amides with Anti-Platelet Aggregation Activity from Piper Taiwanense. Fitoterapia.

[107] Rao V.R.S., Suresh G., Babu K.S., Raju S.S., Vishnu Vardhan M.V.P.S.P.S., Ramakrishna S., Rao J.M. (2011). Novel Dimeric Amide Alkaloids from Piper Chaba Hunter: Isolation, Cytotoxic Activity, and Their Biomimetic Synthesis. Tetrahedron.

[108] Ren J., Xu Y., Huang Q., Yang J., Yang M., Hu K., Wei K. (2015). Chabamide Induces Cell Cycle Arrest and Apoptosis by the Akt/MAPK Pathway and Inhibition of P-Glycoprotein in K562/ADR Cells. Anti Cancer Drugs.

[109] Tabudravu J.N., Jaspars M. (2005). Anticancer Activities of Constituents of Kava (Piper Methysticum). South Pac. J. Nat. Appl. Sci..

[110] Amaral P.D.A., Petrignet J., Gouault N., Agustini T., Lohézic-Ledévéhat F., Cariou A., Grée R., Eifler-Lima V.L., David M. (2009). Synthesis of Novel Kavain-like Derivatives and Evaluation of Their Cytotoxic Activity. J. Braz. Chem. Soc..

[111] Abu N., Akhtar M.N., Yeap S.K., Lim K.L., Ho W.Y., Zulfadli A.J., Omar A.R., Sulaiman M.R., Abdullah M.P., Alitheen N.B. (2014). Flavokawain A Induces Apoptosis in MCF-7 and MDA-MB231 and Inhibits the Metastatic Process In Vitro. PLoS ONE.

[112] Thieury C., Lebouvier N., Le Gu??vel R., Barguil Y., Herbette G., Antheaume C., Hnawia E., Asakawa Y., Nour M., Guillaudeux T. (2017). Mechanisms of Action and Structure-Activity Relationships of Cytotoxic Flavokawain Derivatives. Bioorg. Med. Chem..

[113] Tang Y., Li X., Liu Z., Simoneau A.R., Xie J., Zi X. (2010). Flavokawain B, a Kava Chalcone, Induces Apoptosis via up-Regulation of Death-Receptor 5 and Bim Expression in Androgen Receptor Negative, Hormonal Refractory Prostate Cancer Cell Lines and Reduces Tumor Growth. Int. J. Cancer..

[114] Zhao X., Chao Y.-L., Wan Q.-B., Chen X.-M., Su P., Sun J., Tang Y. (2011). Flavokawain B Induces Apoptosis of Human Oral Adenoid Cystic Cancer ACC-2 Cells via up-Regulation of Bim and down-Regulation of Bcl-2 Expression. Can. J. Physiol. Pharmacol..

[115] Hseu Y.-C., Lee M.-S., Wu C.-R., Cho H.-J., Lin K.-Y., Lai G.-H., Wang S.-Y., Kuo Y.-H., Kumar K.J.S., Yang H.-L. (2012). The Chalcone Flavokawain B Induces G2/M Cell-Cycle Arrest and Apoptosis in Human Oral Carcinoma HSC-3 Cells through the Intracellular ROS Generation and Downregulation of the Akt/P38 MAPK Signaling Pathway. J. Agric. Food Chem..

[116] Eskander R.N., Randall L.M., Sakai T., Guo Y., Hoang B., Zi X. (2012). Flavokawain B, a Novel, Naturally Occurring Chalcone, Exhibits Robust Apoptotic Effects and Induces G2/M Arrest of a Uterine Leiomyosarcoma Cell Linejog. J. Obstet. Gynaecol. Res..

[117] An J., Gao Y., Wang J., Zhu Q., Ma Y., Wu J., Sun J., Tang Y. (2012). Flavokawain B Induces Apoptosis of Non-Small Cell Lung Cancer H460 Cells via Bax-Initiated Mitochondrial and JNK Pathway. Biotechnol. Lett..

[118] Jandial D.D., Krill L.S., Chen L., Wu C., Ke Y., Xie J., Hoang B.H., Zi X. (2017). Induction of G2M Arrest by Flavokawain a, a Kava Chalcone, Increases the Responsiveness of HER2-Overexpressing Breast Cancer Cells to Herceptin. Molecules.

[119] Zi X., Simoneau A.R. (2005). Flavokawain A, a Novel Chalcone from Kava Extract, Induces Apoptosis in Bladder Cancer Cells by Involvement of Bax Protein-Dependent and Mitochondria-Dependent Apoptotic Pathway and Tumor Growth in Mice. Cancer Res..

[120] Phang C.-W., Karsani S.A., Sethi G., Abd Malek S.N. (2016). Flavokawain C Inhibits Cell Cycle and Promotes Apoptosis, Associated with Endoplasmic Reticulum Stress and Regulation of MAPKs and Akt Signaling Pathways in HCT 116 Human Colon Carcinoma Cells. PLoS ONE.

[121] Nurestri S., Malek A., Phang C.W., Ibrahim H., Wahab N.A., Sim K.S. (2011). Phytochemical and Cytotoxic Investigations of Alpinia Mutica Rhizomes. Molecules.

[122] Valadares M.C., de Carvalho I.C.T., de Oliveira Junior L., Vieira M.D.S., Benfica P.L., de Carvalho F.S., Andrade L.V.S., Lima E.M., Kato M.J. (2009). Cytotoxicity and Antiangiogenic Activity of Grandisin. J. Pharm. Pharmacol..

[123] Ferreira I.R.S. (2014). Evaluation of Cytotoxicity of Phytochemicals in V79 Cells and Inhibition of Cell Growth in Human Leukemic Cells.

[124] Vieira M.d.S., de Oliveira V., Lima E.M., Kato M.J., Valadares M.C. (2011). In Vitro Basal Cytotoxicity Assay Applied to Estimate Acute Oral Systemic Toxicity of Grandisin and Its Major Metabolite. Exp. Toxicol. Pathol..

[125] Jiang Z.H., Liu Y.P., Huang Z.H., Wang T.T., Feng X.Y., Yue H., Guo W., Fu Y.H. (2017). Cytotoxic Dihydrobenzofuran Neolignans from Mappianthus Iodoies. Bioorg. Chem..

[126] Sawasdee K., Chaowasku T., Lipipun V., Dufat T.-H., Michel S., Likhitwitayawuid K. (2013). Neolignans from Leaves of Miliusa Mollis. Fitoterapia.

[127] Longato G.B., Rizzo L.Y., De Oliveira Sousa I.M., Tinti S.V., Possenti A., Figueira G.M., Ruiz A.L.T.G., Foglio M.A., De Carvalho J.E. (2011). In Vitro and in Vivo Anticancer Activity of Extracts, Fractions, and Eupomatenoid-5 Obtained from Piper Regnellii Leaves. Planta Med..

[128] Bley K., Boorman G., Mohammad B., McKenzie D., Babbar S. (2012). A Comprehensive Review of the Carcinogenic and Anticarcinogenic Potential of Capsaicin. Toxicol. Pathol..

[129] De-Sá-Júnior P.L., Pasqualoto K.F.M., Ferreira A.K., Tavares M.T., Damião M.C.F.C.B., De Azevedo R.A., Câmara D.A.D., Pereira A., De Souza D.M., Parise Filho R. (2013). RPF101, A New Capsaicin-like Analogue, Disrupts the Microtubule Network Accompanied by Arrest in the G2/M Phase, Inducing Apoptosis and Mitotic Catastrophe in the MCF-7 Breast Cancer Cells. Toxicol. Appl. Pharmacol..

[130] Damião M.C.F.C.B., Pasqualoto K.F.M., Ferreira A.K., Teixeira S.F., Azevedo R.A., Barbuto J.A.M., Palace-Berl F., Franchi-Junior G.C., Nowill A.E., Tavares M.T. (2014). Novel Capsaicin Analogues as Potential Anticancer Agents: Synthesis, Biological Evaluation, and In Silico Approach. Arch. Der Pharm..

[131] Ferreira A.K., Tavares M.T., Pasqualoto K.F.M., de Azevedo R.A., Teixeira S.F., Ferreira-Junior W.A., Bertin A.M., de-Sá-Junior P.L., Barbuto J.A.M., Figueiredo C.R. (2015). RPF151, a Novel Capsaicin-like Analogue: In Vitro Studies and in Vivo Preclinical Antitumor Evaluation in a Breast Cancer Model. Tumor Biol..

[132] Tavares M.T. (2014). Novel Anticancer Candidates: Synthesis and Antitumor Activity of Capsaicin-like Sulfonate and Sulfonamide Analogues.

[133] Batista Fernandes T., Alexandre de Azevedo R., Yang R., Fernandes Teixeira S., Henrique Goulart Trossini G., Alexandre Marzagao Barbuto J., Kleber Ferreira A., Parise Filho R. (2018). Arylsulfonylhydrazone Induced Apoptosis in MDA-MB-231 Breast Cancer Cells. Lett. Drug Des. Discov..

[134] Pereira G.J.V., Tavares M.T., Azevedo R.A., Martins B.B., Cunha M.R., Bhardwaj R., Cury Y., Zambelli V.O., Barbosa E.G., Hediger M.A. (2019). Capsaicin-like Analogue Induced Selective Apoptosis in A2058 Melanoma Cells: Design, Synthesis and Molecular Modeling. Bioorg. Med. Chem..

[135] Kotake-Nara E., Kushiro M., Zhang H., Sugawara T., Miyashita K., Nagao A. (2001). Carotenoids Affect Proliferation of Human Prostate Cancer Cells. J. Nutr..

[136] Molnár J., Serly J., Pusztai R., Vincze I., Molnár P., Horváth G., Deli J., Maoka T., Zalatnai A., Tokuda H. (2012). Putative Supramolecular Complexes Formed by Carotenoids and Xanthophylls with Ascorbic Acid to Reverse Multidrug Resistance in Cancer Cells. Anticancer Res..

[137] Zhang L., Shamaladevi N., Jayaprakasha G.K., Patil B.S., Lokeshwar B.L. (2015). Polyphenol-Rich Extract of *Pimenta Dioica* Berries (Allspice) Kills Breast Cancer Cells by Autophagy and Delays Growth of Triple Negative Breast Cancer in Athymic Mice. Oncotarget.

[138] De Oliveira Chaves M.C., de Oliveira A.H., de Oliveira Santos B.V. (2006). Aristolactams from Piper Marginatum Jacq (Piperaceae). Biochem. Syst. Ecol..

[139] Kim K.H., Choi J.W., Choi S.U., Ha S.K., Kim S.Y., Park H.-J., Lee K.R. (2011). The Chemical Constituents of Piper Kadsura and Their Cytotoxic and Anti-Neuroinflammtaory Activities. J. Enzym. Inhib. Med. Chem..

[140] Shibutani S., Dong H., Suzuki N., Ueda S., Miller F., Grollman A.P. (2007). Selective Toxicity of Aristolochic Acids I and II. Drug Metab. Dispos..

[141] Michl J., Ingrouille M.J., Simmonds M.S.J., Heinrich M. (2014). Naturally Occurring Aristolochic Acid Analogues and Their Toxicities. Nat. Prod. Rep..

[142] Asha K.N., Chowdhury R., Hasan C.M., Rashid M.A. (2003). Antibacterial Activity and Cytotoxicity of Extractives from Uvaria Hamiltonii Stem Bark. Fitoterapia.

[143] De Moraes J., Nascimento C., Yamaguchi L.F., Kato M.J., Nakano E. (2012). Schistosoma Mansoni: In Vitro Schistosomicidal Activity and Tegumental Alterations Induced by Piplartine on Schistosomula. Exp. Parasitol..

[144] Kumar J.U., Shankaraiah G., Kumar R.S.C., Pitke V.V., Rao T., Poornima B., Babu K.S., Sreedhar A.S. (2013). Synthesis, Anticancer, and Antibacterial Activities of Piplartine Derivatives on Cell Cycle Regulation and Growth Inhibition. J. Asian Nat. Prod. Res..

[145] Da Nóbrega F., Ozdemir O., Nascimento Sousa S., Barboza J., Turkez H., de Sousa D. (2018). Piplartine Analogues and Cytotoxic Evaluation against Glioblastoma. Molecules.

[146] Liu J.M., Pan F., Li L., Liu Q.R., Chen Y., Xiong X.X., Cheng K., Yu S.B., Shi Z., Yu A.C.-H. (2013). Piperlongumine Selectively Kills Glioblastoma Multiforme Cells via Reactive Oxygen Species Accumulation Dependent JNK and P38 Activation. Biochem. Biophys. Res. Commun..

[147] Niu M., Xu X., Shen Y., Yao Y., Qiao J., Zhu F., Zeng L., Liu X., Xu K. (2015). Piperlongumine Is a Novel Nuclear Export Inhibitor with Potent Anticancer Activity. Chem. Biol. Interact..

[148] Piska K., Gunia-Krzyżak A., Koczurkiewicz P., Wójcik-Pszczoła K., Pękala E. (2018). Piperlongumine (Piplartine) as a Lead Compound for Anticancer Agents–Synthesis and Properties of Analogues: A Mini-Review. Eur. J. Med. Chem..

[149] Smith R.M. (1979). Pipermethystine, a Novel Pyridone Alkaloid from Piper Methysticum. Tetrahedron.

[150] Dunstan W.R., Garnett H. (1895). XII.—The Constituents of Piper Ovatum. J. Chem. Soc. Trans..

[151] Pandey S., Ovadje P.U. (2014). Long Pepper Extract an Effective Anticancer Treatment. U.S. Patent.

[152] Bezerra D.P., Pessoa C., de Moraes M.O., de Alencar N.M.N., Mesquita R.O., Lima M.W., Alves A.P.N.N., Pessoa O.D.L., Chaves J.H., Silveira E.R. (2008). In Vivo Growth Inhibition of Sarcoma 180 by Piperlonguminine, an Alkaloid Amide from the Piper Species. J. Appl. Toxicol..

[153] Miranda J.E., Navickiene H.M.D., Nogueira-Couto R.H., De Bortoli S.A., Kato M.J., Bolzani V.D.S., Furlan M. (2003). Susceptibility of Apis Mellifera (Hymenoptera: Apidae) to Pellitorine, an Amide Isolated from Piper Tuberculatum (Piperaceae). Apidologie.

[154] Damanhouri Z.A., Ahmad A. (2014). A Review on Therapeutic Potential of Piper Nigrum L. (Black Pepper): The King of Spices. Med. Aromat. Plants.

[155] Qu H., Lv M., Xu H. (2015). Piperine: Bioactivities and Structural Modifications. Mini Rev. Med. Chem..

[156] Jong-Woong A., Mi-Ja A., Ok-Pyo Z., Eun-Joo K., Sueg-Geun L., Hyung J.K., Kubo I. (1992). Piperidine Alkaloids from Piper Retrofractum Fruits. Phytochemistry.

[157] Shoji N., Umeyama A., Saito N., Takemoto T., Kajiwara A., Ohizumi Y. (1986). Dehydropipernonaline, an Amide Possessing Coronary Vasodilating Activity, Isolated from Piper Iongum L.. J. Pharm. Sci..

[158] Tabuneng W., Bando H., Amiya T. (1983). Studies on the Constituents of the Crude Drug “Piperis Longi Fructus.” On the Alkaloids of Fruits of Piper Longum L.. Chem. Pharm. Bull..

[159] Lee W., Kim K.-Y., Yu S.-N., Kim S.-H., Chun S.-S., Ji J.-H., Yu H.-S., Ahn S.-C. (2013). Pipernonaline from Piper Longum Linn. Induces ROS-Mediated Apoptosis in Human Prostate Cancer PC-3 Cells. Biochem. Biophys. Res. Commun..

[160] Lee F.-P., Chen Y.-C., Chen J.-J., Tsai I.-L., Chen I.-S. (2004). Cyclobutanoid Amides from Piper Arborescens. Helv. Chim. Acta.

[161] Gutekunst W.R., Baran P.S. (2011). Total Synthesis and Structural Revision of the Piperarborenines via Sequential Cyclobutane C–H Arylation. J. Am. Chem. Soc..

[162] Frébault F., Maulide N. (2012). Total Synthesis and Structural Revision of the Piperarborenines: When Photochemistry Meets C—H Activation. Angew. Chem. Int. Ed..

[163] Panish R.A., Chintala S.R., Fox J.M. (2016). A Mixed-Ligand Chiral Rhodium(II) Catalyst Enables the Enantioselective Total Synthesis of Piperarborenine B. Angew. Chem. Int. Ed..

[164] Hu J.-L., Feng L.-W., Wang L., Xie Z., Tang Y., Li X. (2016). Enantioselective Construction of Cyclobutanes: A New and Concise Approach to the Total Synthesis of (+)-Piperarborenine B. J. Am. Chem. Soc..

[165] Rao V.R.S., Suresh Kumar G., Sarma V.U.M., Satyanarayana Raju S., Hari Babu K., Suresh Babu K., Hari Babu T., Rekha K., Rao J.M. (2009). Chabamides F and G, Two Novel Dimeric Alkaloids from the Roots of Piper Chaba Hunter. Tetrahedron Lett..

[166] Da Silva R.V., Debonsi Navickiene H.M., Kato M.J., Bolzani V.D.S., Méda C.I., Young M.C.M., Furlan M. (2002). Antifungal Amides from Piper Arboreum and Piper Tuberculatum. Phytochemistry.

[167] Hu K., Yang M., Xu Y., Wei K., Ren J. (2015). Cell Cycle Arrest, Apoptosis, and Autophagy Induced by Chabamide in Human Leukemia Cells. Chin. Herb. Med..

[168] Steiner G.G. (2000). The Correlation between Cancer Incidence and Kava Consumption. Hawaii Med. J..

[169] Li X., Liu Z., Xu X., Blair C.A., Sun Z., Xie J., Lilly M.B., Zi X. (2012). Kava Components Down-Regulate Expression of AR and AR Splice Variants and Reduce Growth in Patient-Derived Prostate Cancer Xenografts in Mice. PLoS ONE.

[170] Tang J., Dunlop R.A., Rowe A., Rodgers K.J., Ramzan I. (2010). Kavalactones Yangonin and Methysticin Induce Apoptosis in Human Hepatocytes (HepG2) In Vitro. Phytother. Res..

[171] Zou L., Henderson G.L., Harkey M.R., Sakai Y., Li A. (2004). Effects of Kava (Kava-Kava, ’Awa, Yaqona, Piper Methysticum) on c-DNA-Expressed Cytochrome P450 Enzymes and Human Cryopreserved Hepatocytes. Phytomed. Int. J. Phytother. Phytopharm..

[172] Flores N., Cabrera G., Jiménez I., Piñero J., Giménez A., Bourdy G., Cortés-Selva F., Bazzocchi I. (2007). Leishmanicidal Constituents from the Leaves of *Piper Rusbyi*. Planta Med..

[173] Dos Santos R.A., Ramos C.S., Young M.C.M., Pinheiro T.G., Amorim A.M., Kato M.J., Batista R. (2013). Antifungal Constituents from the Roots of *Piper Dilatatum* Rich. J. Chem..

[174] Sakai T., Eskander R.N., Guo Y., Kim K.J., Mefford J., Hopkins J., Bhatia N.N., Zi X., Hoang B.H. (2012). Flavokawain B, a Kava Chalcone, Induces Apoptosis in Synovial Sarcoma Cell Lines. J. Orthop. Res. Off. Publ. Orthop. Res. Soc..

[175] Phang C.-W., Karsani S., Abd Malek S. (2017). Induction of Apoptosis and Cell Cycle Arrest by Flavokawain C on HT-29 Human Colon Adenocarcinoma via Enhancement of Reactive Oxygen Species Generation, Upregulation of P21, P27, and Gadd153, and Inactivation of Inhibitor of Apoptosis Proteins. Pharmacogn. Mag..

[176] Lina E., Lin W.-H., Wang S.-Y., Chen C.-S., Liao J.-W., Chang H.-W., Chen S.-C., Lin K.-Y., Wang L., Yangh H.-L. (2012). Flavokawain B Inhibits Growth of Human Squamous Carcinoma Cells: Involvement of Apoptosis and Cell Cycle Dysregulation in Vitro and in Vivo. J. Nutr. Biochem..

[177] Ji T., Lin C., Krill L.S., Eskander R., Guo Y., Zi X., Hoang B.H. (2013). Flavokawain B, a Kava Chalcone, Inhibits Growth of Human Osteosarcoma Cells through G2/M Cell Cycle Arrest and Apoptosis. Mol. Cancer.

[178] Abu N., Akhtar M.N., Yeap S.K., Lim K.L., Ho W.Y., Abdullah M.P., Ho C.L., Omar A.R., Ismail J., Alitheen N.B. (2016). Flavokawain B Induced Cytotoxicity in Two Breast Cancer Cell Lines, MCF-7 and MDA-MB231 and Inhibited the Metastatic Potential of MDA-MB231 via the Regulation of Several Tyrosine Kinases In Vitro. BMC Complement. Altern. Med..

[179] Martins R.C.C., Latorre L.R., Sartorelli P., Kato M.J. (2000). Phenylpropanoids and Tetrahydrofuran Lignans from Piper Solmsianum. Phytochemistry.

[180] Ramos C.S., Linnert H.V., de Moraes M.M., do Amaral J.H., Yamaguchi L.F., Kato M.J. (2017). Configuration and Stability of Naturally Occurring All-Cis-Tetrahydrofuran Lignans from Piper Solmsianum. RSC Adv..

[181] Barth T., Habenschus M.D., Lima Moreira F., Ferreira L.D.S., Lopes N.P., Moraes de Oliveira A.R. (2015). In Vitro Metabolism of the Lignan (−)-Grandisin, an Anticancer Drug Candidate, by Human Liver Microsomes. Drug Test. Anal..

[182] Messiano G.B., Santos R.A.D.S., Ferreira L.D.S., Simões R.A., Jabor V.A.P., Kato M.J., Lopes N.P., Pupo M.T., de Oliveira A.R.M. (2013). In Vitro Metabolism Study of the Promising Anticancer Agent the Lignan (-)-Grandisin. J. Pharm. Biomed. Anal..

[183] Cortez A.P., Menezes E.G.P., Benfica P.L., Santos A.P.d., Cleres L.M., Ribeiro H.D.O., Lima E.M., Kato M.J., Valadares M.C. (2017). Grandisin Induces Apoptosis in Leukemic K562 Cells. Braz. J. Pharm. Sci..

[184] Stecanella L.A., Taveira S.F., Marreto R.N., Valadares M.C., Vieira M.d.S., Kato M.J., Lima E.M. (2013). Development and Characterization of PLGA Nanocapsules of Grandisin Isolated from Virola Surinamensis: In Vitro Release and Cytotoxicity Studies. Braz. J. Pharmacogn..

[185] Ma Y., Han G.Q., Wang Y.Y. (1993). PAF Antagonistic Benzofuran Neolignans from Piper Kadsura. Acta Pharm. Sin..

[186] Chauret D.C., Bernard C.B., Arnason J.T., Durst T., Krishnamurty H.G., Sanchez-Vindas P., Moreno N., San Roman L., Poveda L. (1996). Insecticidal Neolignans from Piper Decurrens. J. Nat. Prod..

[187] Zheng S., Yu W., Xu M., Che C. (2003). First Synthesis of Naturally Occurring (±)-\textlessi\textgreaterepi\textless/I\textgreater-Conocarpan. Tethrahedron Lett..

[188] Campos M.P., Cechinel Filho V., Silva R.Z., Yunes R.A., Monache F.D., Cruz A.B. (2007). Antibacterial Activity of Extract, Fractions and Four Compounds Extracted from Piper Solmsianum C. DC. Var. Solmsianum (Piperaceae). Z. Fur Nat. Sect. C J. Biosci..

[189] Johann S., Cota B.B., Souza-Fagundes E.M., Pizzolatti M.G., Resende M.A., Zani C.L. (2009). Antifungal Activities of Compounds Isolated from Piper Abutiloides Kunth. Mycoses.

[190] Moreira D.D.L., de Paiva R.A., Marques A.M., Borges R.M., Barreto A.L.S., Curvelo J.A.D.R., Cavalcanti J.F., Romanos M.T.V., Romanos M.T.V., Soares  R.M.D.A. (2016). Bioactive Neolignans from the Leaves of Piper Rivinoides Kunth (Piperaceae). Rec. Nat. Prod..

[191] Rimando A.M., Pezzuto J.M., Farnsworth N.R., Santisuk T., Reutrakul V. (1994). Revision of the NMR Assignments of Pterostlbene and of Dihydrodehydrodiconiferyl Alcohol: Cytotoxic Constituents from Anogeissus Acuminata. Nat. Prod. Lett..

[192] Longato G.B., Fiorito G.F., Vendramini-Costa D.B., Sousa I.M.D.O., Tinti S.V., Ruiz A.L.T.G., de Almeida S.M.V., Padilha R.J.R., Foglio M.A., de Carvalho J.E. (2015). Different Cell Death Responses Induced by Eupomatenoid-5 in MCF-7 and 786-0 Tumor Cell Lines. Toxicol. Vitr..

[193] Gibbs H.A.A., O’Garro L.W. (2004). Capsaicin Content of West Indies Hot Pepper Cultivars Using Colorimetric and Chromatographic Techniques. HortScience.

[194] Sanatombi K., Sharma G.J. (2008). Capsaicin Content and Pungency of Different Capsicum Spp. Cultivars. Not. Bot. Horti Agrobot. Cluj-Napoca.

[195] Thapa B., Skalko-Basnet N., Takano A., Masuda K., Basnet P. (2009). High-Performance Liquid Chromatography Analysis of Capsaicin Content in 16 Capsicum Fruits from Nepal. J. Med. Food.

[196] Yaldiz G., Ozguven M., Sekeroglu N. (2010). Variation in Capsaicin Contents of Different Capsicum Species and Lines by Varying Drying Parameters. Ind. Crop. Prod..

[197] Hayman M., Kam P.C.A. (2008). Capsaicin: A Review of Its Pharmacology and Clinical Applications. Curr. Anaesth. Crit. Care.

[198] Luo X.-J.J., Peng J., Li Y.-J.J. (2011). Recent Advances in the Study on Capsaicinoids and Capsinoids. Eur. J. Pharmacol..

[199] Sánchez A.M., Sánchez M.G., Malagarie-Cazenave S., Olea N., Díaz-Laviada I. (2006). Induction of Apoptosis in Prostate Tumor PC-3 Cells and Inhibition of Xenograft Prostate Tumor Growth by the Vanilloid Capsaicin. Apoptosis.

[200] De Lourdes Reyes-Escogido M., Gonzalez-Mondragon E.G., Vazquez-Tzompantzi E. (2011). Chemical and Pharmacological Aspects of Capsaicin. Molecules.

[201] Ryu H.C., Seo S., Kim M.S., Kim M.Y., Kim H.O., Ann J., Tran P.T., Hoang V.H., Byun J., Cui M. (2014). 2-Aryl Substituted Pyridine C-Region Analogues of 2-(3-Fluoro-4-Methylsulfonylaminophenyl)Propanamides as Highly Potent TRPV1 Antagonists. Bioorg. Med. Chem. Lett..

[202] Darré L., Domene C. (2015). Binding of Capsaicin to the TRPV1 Ion Channel. Mol. Pharm..

[203] Yang F., Zheng J. (2017). Understand Spiciness: Mechanism of TRPV1 Channel Activation by Capsaicin. Protein Cell.

[204] Yang F., Xiao X., Cheng W., Yang W., Yu P., Song Z., Yarov-Yarovoy V., Zheng J. (2015). Structural Mechanism Underlying Capsaicin Binding and Activation of the TRPV1 Ion Channel. Nat. Chem. Biol..

[205] Lee J.H., Lee Y., Ryu H., Kang D.W., Lee J., Lazar J., Pearce L.V., Pavlyukovets V.A., Blumberg P.M., Choi S. (2011). Structural Insights into Transient Receptor Potential Vanilloid Type 1 (TRPV1) from Homology Modeling, Flexible Docking, and Mutational Studies. J. Comput. Aided Mol. Des..

[206] Gao Y., Cao E., Julius D., Cheng Y. (2016). TRPV1 Structures in Nanodiscs Reveal Mechanisms of Ligand and Lipid Action. Nature.

[207] Cui J., Bian J.S., Kagan A., McDonald T.V. (2002). CaT1 Contributes to the Stores-Operated Calcium Current in Jurkat T-Lymphocytes. J. Biol. Chem..

[208] Pérez De Vega M.J., Gómez-Monterrey I., Ferrer-Montiel A., González-Muñiz R. (2016). Transient Receptor Potential Melastatin 8 Channel (TRPM8) Modulation: Cool Entryway for Treating Pain and Cancer. J. Med. Chem..

[209] Chow J., Norng M., Zhang J., Chai J. (2007). TRPV6 Mediates Capsaicin-Induced Apoptosis in Gastric Cancer Cells—Mechanisms behind a Possible New “Hot” Cancer Treatment. Biochim. Et Biophys. Acta (Bba) Mol. Cell Res..

[210] Cunha M.R., Bhardwaj R., Carrel A.L., Lindinger S., Romanin C., Parise-Filho R., Hediger M.A., Reymond J.-L. (2020). Natural Product Inspired Optimization of a Selective TRPV6 Calcium Channel Inhibitor. Rsc Med. Chem..

[211] Lau J.K., Brown K.C., Dom A.M., Witte T.R., Thornhill B.A., Crabtree C.M., Perry H.E., Brown J.M., Ball J.G., Creel R.G. (2014). Capsaicin Induces Apoptosis in Human Small Cell Lung Cancer via the TRPV6 Receptor and the Calpain Pathway. Apoptosis.

[212] Ip S.W., Lan S.H., Huang A.C., Yang J.S., Chen Y.Y., Huang H.Y., Lin Z.P., Hsu Y.M., Yang M.D., Chiu C.F. (2012). Capsaicin Induces Apoptosis in SCC-4 Human Tongue Cancer Cells through Mitochondria-Dependent and -Independent Pathways. Environ. Toxicol..

[213] Ito K., Nakazato T., Yamato K., Miyakawa Y., Yamada T., Hozumi N., Segawa K., Ikeda Y., Kizaki M. (2004). Induction of Apoptosis in Leukemic Cells by Homovanillic Acid Derivative, Capsaicin, through Oxidative Stress: Implication of Phosphorylation of P53 at Ser-15 Residue by Reactive Oxygen Species. Cancer Res..

[214] Tavares M.T., Pasqualoto K.F.M., van de Streek J., Ferreira A.K., Azevedo R.A., Damião M.C.F.C.B., Rodrigues C.P., de-Sá-Júnior P.L., Barbuto J.A.M., Parise-Filho R. (2015). Synthesis, Characterization, in Silico Approach and in Vitro Antiproliferative Activity of RPF151, a Benzodioxole Sulfonamide Analogue Designed from Capsaicin Scaffold. J. Mol. Struct..

[215] Cunha M.R., Tavares M.T., Carvalho C.F., Silva N.A.T., Souza A.D.F., Pereira G.J.V., Ferreira F.F., Parise-Filho R. (2016). Environmentally Safe Condition for the Synthesis of Aryl and Alkyl Sulfonyl Hydrazones via One-Pot Reaction. Acs Sustain. Chem. Eng. Sustain. Chem. Eng..

[216] Ha S.-H., Kim J.-B., Park J.-S., Lee S.-W., Cho K.-J. (2007). A Comparison of the Carotenoid Accumulation in Capsicum Varieties That Show Different Ripening Colours: Deletion of the Capsanthin-Capsorubin Synthase Gene Is Not a Prerequisite for the Formation of a Yellow Pepper. J. Exp. Bot..

[217] Narisawa T., Fukaura Y., Hasebe M., Nomura S., Oshima S., Inakuma T. (2000). Prevention of N-Methylnitrosourea-Induced Colon Carcinogenesis in Rats by Oxygenated Carotenoid Capsanthin and Capsanthin-Rich Paprika Juice. Proc. Soc. Exp. Biol. Med..

