# Supplementary material for: Peppers: A “Hot” Natural Source for Antitumor Compounds
Source: Molecules. 2021 Mar 10;26(6):1521. doi: 10.3390/molecules26061521 (PMC8002096; doi:10.3390/molecules26061521)
Supplement: Supplementary file 1 [file molecules-26-01521-s001.pdf]

Supporting Information

Peppers: A "Hot" Natural Source for Antitumor Compounds

Micael Rodrigues Cunha [1,3], Maurício Temotheo Tavares [2,3], Thais Batista Fernandes [3] and Roberto Parise-Filho [3*]

[1] Center of Medicinal Chemistry, Dr. André Tosello Avenue, 550, 13083-886 Campinas - SP, Brazil; micaelrc@unicamp.br
[2] Department of Molecular Medicine, The Scripps Research Institute, Jupiter, FL 33458, United States; mttavares@scripps.edu
[3] Laboratory of Design and Synthesis of Bioactive Substances, Department of Pharmacy, University of São Paulo, Prof. Lineu Prestes Avenue 580, Bl.13, 05508-900 Butantã - SP, Brazil; thaisbf@alumni.usp.br
* Correspondence: roberto.parise@usp.br; Tel.: +55-11-3091-3687

Table 1. Description of cancer cell lines from table 1.

Cell line	Species	Disease
3T3-A31	*Mus musculus*	fibroblast, normal
518A2	*Homo sapiens*	melanoma
786-0	*Homo sapiens*	renal adenocarcinoma
A2058	*Homo sapiens*	melanoma
A2780	*Homo sapiens*	ovarian carcinoma
A375	*Homo sapiens*	malignant melanoma
A549	*Homo sapiens*	lung adenocarcinoma
ACC-2	*Homo sapiens*	human papillomavirus-related endocervical adenocarcinoma
AsPC1	*Homo sapiens*	pancreas adenocarcinoma
B16F10	*Mus musculus*	melanoma
BxPC3	*Homo sapiens*	pancreas adenocarcinoma
CaCo-2	*Homo sapiens*	colorectal adenocarcinoma
Cal-27	*Homo sapiens*	squamous cell carcinoma
CaSKi	*Homo sapiens*	epidermoid carcinoma
CCD-18Co	*Homo sapiens*	fibroblast, normal
CEM	*Homo sapiens*	acute lymphoblastic leukemia
CNE	*Homo sapiens*	nasopharyngeal carcinoma
COLO-205	*Homo sapiens*	Dukes' type D, colorectal adenocarcinoma
DU-145	*Homo sapiens*	prostate carcinoma
EAT	*Mus musculus*	breast adenocarcinoma
EJ	*Homo sapiens*	endometrial adenocarcinoma
GBM10	*Homo sapiens*	glioblastoma
H460	*Homo sapiens*	large cell lung carcinoma
HaCaT	*Homo sapiens*	immortalized skin keratinocytes
HCT116	*Homo sapiens*	colorectal carcinoma
HCT15	*Homo sapiens*	Dukes' type C, colorectal adenocarcinoma
HCT8	*Homo sapiens*	ileocecal colorectal adenocarcinoma
HeLa	*Homo sapiens*	epithelial adenocarcinoma
HepG2	*Homo sapiens*	hepatocellular carcinoma
HL60	*Homo sapiens*	acute myeloid leukemia

HSC-3	*Homo sapiens*	tongue squamous carcinoma
HT1080	*Homo sapiens*	fibrosarcoma
HT-29	*Homo sapiens*	colorectal adenocarcinoma
HuH7	*Homo sapiens*	hepatocellular carcinoma
IMR-32	*Homo sapiens*	neuroblastoma
JURKAT	*Homo sapiens*	acute T cell leukemia
K-562	*Homo sapiens*	chronic myelogenous leukemia
KB	*Homo sapiens*	carcinoma
L-02	*Homo sapiens*	immortalized fetal hepatocyte
L1210	*Mus musculus*	lymphocytic leukemia
L5178Y	*Mus musculus*	lymphoma
LAPC4	*Homo sapiens*	prostate carcinoma
LNCaP	*Homo sapiens*	prostate adenocarcinoma
MCF-10A	*Homo sapiens*	epithelial breast, normal
MCF-12A	*Homo sapiens*	immortalized epithelial breast
MCF-7	*Homo sapiens*	breast adenocarcinoma
MDA-MB-231	*Homo sapiens*	breast metastatic adenocarcinoma
MDA-MB-468	*Homo sapiens*	breast metastatic adenocarcinoma
MOLT-4	*Homo sapiens*	acute lymphoblastic leukemia
MRC-5	*Homo sapiens*	lung, normal
NCI-H460	*Homo sapiens*	large cell lung carcinoma
NCI-H727	*Homo sapiens*	lung carcinoid
OVCAR-3	*Homo sapiens*	ovary adenocarcinoma
P-388	*Mus musculus*	lymphoma
PC-3	*Homo sapiens*	grade IV, prostate adenocarcinoma
RL	*Homo sapiens*	non-Hodgkin's lymphoma
RT-4	*Homo sapiens*	urinary bladder transitional cell papilloma
SF188	*Homo sapiens*	glioblastoma
SGC-7901	*Homo sapiens*	human papillomavirus-related endocervical adenocarcinoma
SKBR3	*Homo sapiens*	breast metastatic adenocarcinoma
SK-LMS-1	*Homo sapiens*	leiomyosarcoma
SK-MEL-2	*Homo sapiens*	malignant melanoma
SK-MEL-25	*Homo sapiens*	malignant melanoma
SK-MEL-28	*Homo sapiens*	cutaneous melanoma
SK-OV-3	*Homo sapiens*	ovary adenocarcinoma
SMMC-7721	*Homo sapiens*	human papillomavirus-related endocervical adenocarcinoma
SW-480	*Homo sapiens*	Dukes' type B, colorectal adenocarcinoma
SW-620	*Homo sapiens*	Dukes' type C, colorectal adenocarcinoma
T98G	*Homo sapiens*	glioblastoma multiforme
U-87	*Homo sapiens*	glioblastoma
U937	*Homo sapiens*	histiocytic lymphoma
UACC-62	*Homo sapiens*	melanoma
V79	*Cricetulus griseus*	fibroblast, normal
WI38	*Homo sapiens*	fibroblast, normal
ZR-75-30	*Homo sapiens*	breast ductal carcinoma
